# ABCA1 acts as a protective modulator in amyotrophic lateral sclerosis

**DOI:** 10.1016/j.isci.2025.114320

**Published:** 2025-12-03

**Authors:** Qiang Li, Ge Zhang, Honglin Zheng, Taiqi Zhao, Hang Zhang, Yaochong Zhang, Haiyang Luo, Yuming Xu

**Affiliations:** 1 Department of Neurology, The First Affiliated Hospital of Zhengzhou University, Zhengzhou University, Zhengzhou, Henan 450052, China; 2 Department of Cardiology, The First Affiliated Hospital of Zhengzhou University, Henan Key Laboratory of Chronic Disease Prevention and Therapy & Intelligent Health Management, Tianjian Laboratory of Advanced Biomedical Sciences, Academy of Medical Sciences, Zhengzhou University, Zhengzhou, Henan 450001, China; 3 NHC Key Laboratory of Prevention and treatment of Cerebrovascular Disease, Zhengzhou, Henan 450052, China; 4 Henan Key Laboratory of Cerebrovascular Diseases, Zhengzhou University, Zhengzhou, Henan 450052, China

**Keywords:** Health sciences

## Abstract

Amyotrophic lateral sclerosis (ALS) is a progressive motor neuron disease lacking reliable biomarkers and effective therapeutic targets. We performed an integrative multiscale analysis combining global epidemiology, whole-blood transcriptomics, machine learning, and Mendelian randomization (MR). We developed a nine-gene diagnostic signature (AUC = 0.75 in external validation) and identified ATP-binding cassette transporter A1 (*ABCA1*) as a central feature. MR analyses supported a protective causal relationship between increased ABCA1 expression and reduced ALS risk (OR = 0.93, *p* = 0.02). We validated this at the protein level, finding serum ABCA1 significantly elevated in an in-house ALS cohort (*p* = 0.006) and correlated with metabolic parameters (BMI and LDL). Spatiotemporal profiling confirmed ABCA1 upregulation in ALS patient blood and spinal cords, and progressive upregulation in ALS model mice. Collectively, we validated a diagnostic signature and identified ABCA1 as a protective, compensatory biomarker in ALS, emphasizing the link between metabolic adaptation and neurodegeneration.

## Highlights

• A nine-gene diagnostic signature is developed and validated for ALS diagnosis
• Mendelian randomization supports a causal protective role for ABCA1 in ALS
• Serum ABCA1 is elevated in ALS and correlates with BMI and LDL levels
• ABCA1 exhibits spatial and temporal heterogeneity of expression in ALS

## Graphical abstract

**Figure:**
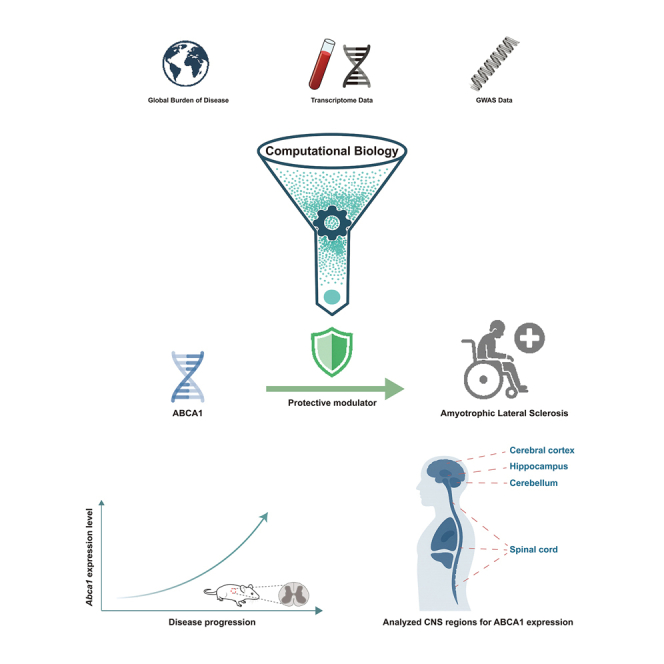

## Introduction

Amyotrophic lateral sclerosis is a progressive neurodegenerative disorder characterized by the selective loss of upper and lower motor neurons, leading to paralysis and death typically within 3–5 years of symptom onset.^1^ According to the Global Burden of Disease 2016 data, the age-standardized incidence and prevalence of motor neuron diseases have shown an increasing trend since 1990, particularly in regions with higher SDI levels, likely reflecting improved diagnostic awareness and region-specific environmental or lifestyle risk factors.^2^ Within this disease spectrum, ALS constitutes the predominant subtype, accounting for approximately 80–90% of all motor neuron disease cases.^1^^,^^2^ Despite its relatively low incidence of approximately 1–3 cases per 100,000 person-years, ALS has a disproportionate impact because of its rapid progression and lack of effective treatments.^1^^,^^3^ The growing global burden of ALS underscores the urgent need for biomarkers that can facilitate earlier diagnosis, track disease progression, and reveal novel therapeutic targets.

Emerging evidence suggests that ALS is a clinically heterogeneous neurodegenerative disease underpinned by a mosaic of high-penetrance mutations and polygenic background risk.^4^^,^^5^^,^^6^^,^^7^ Although variants in *SOD1*, *C9orf72,* and a handful of other genes explain about 10% of cases, the remaining majority carry many low-effect alleles whose collective impact on molecular networks is still poorly resolved.^8^^,^^9^ Genome-wide association studies (GWASs) have identified dozens of loci; however, translating these statistical hits into mechanistic insights and identifying clinically actionable biomarkers remains challenging.

High-throughput transcriptomics provides a crucial bridge between genetic variation and phenotypic manifestation. However, cross-study inconsistencies, arising from platform differences and limited sample sizes, often yield fragmented results. Integrating multiple independent transcriptomic datasets from the Gene Expression Omnibus can mitigate these biases, enhance reproducibility, and improve statistical power.^10^^,^^11^ Systems-level integration further addresses this limitation: weighted gene co-expression network analysis (WGCNA) can uncover biologically coherent modules, while complementary machine learning (ML) algorithms distill reproducible diagnostic signatures. Integrating these molecular features with MR analyses enables causal inference, and protein-level validation further establishes translational relevance. Here, we performed a comprehensive, multiscale systems analysis to identify protective biomarkers and therapeutic targets in ALS. Our analytical framework encompassed (i) epidemiological assessment of global ALS trends; (ii) WGCNA of peripheral blood transcriptomes to identify ALS-associated modules; (iii) differential expression and pathway enrichment analyses to highlight dysregulated genes and biological pathways; (iv) application of machine learning algorithms, including random forest (RF), least absolute shrinkage and selection operator (LASSO), and support vector machine-recursive feature elimination (SVM-RFE), to derive a diagnostic gene signature; (v) validation of this signature’s predictive performance across independent cohorts; (vi) MR analyses to evaluate causal relationships between the expression of diagnostic signature genes and ALS risk; (vii) biochemical validation of ABCA1, the MR-supported causal and protective candidate, in an independent in-house serum cohort, followed by integrative analyses across multiple Gene Expression Omnibus (GEO) datasets to comprehensively delineate its spatial and temporal expression heterogeneity in ALS. *ABCA1* consistently emerged as the convergent hub across co-expression, machine learning, and causal inference analyses, underscoring its potential causal and protective relevance to ALS and establishing it as a focal candidate for downstream validation. Cholesterol metabolism dysregulation has been implicated in ALS pathogenesis, yet its causal direction remains debated.^12^^,^^13^^,^^14^^,^^15^^,^^16^ And *ABCA1* functions as an inducible cholesterol efflux transporter and immunomodulator.^17^^,^^18^^,^^19^ It is widely expressed in peripheral immune cells (e.g., monocytes and macrophages) and in the CNS (astrocytes and microglia), where it responds to sterol and inflammatory signals.^20^^,^^21^^,^^22^ Interestingly, the *ABCA1*-liver X receptor (LXR) axis offers a mechanistic link between cholesterol handling and neuroprotection: LXR activation upregulates *ABCA1*, which transfers excess intracellular cholesterol to apoA-I, initiates reverse-cholesterol transport and maintains lipid homeostasis.^17^^,^^23^ Efficient efflux prevents cholesterol accumulation in microglia and macrophages, thereby dampening proinflammatory signaling,^24^ whereas *ABCA1* deficiency produces lipid-laden phagocytes and exacerbates tissue damage.^18^^,^^24^ Although *ABCA1* has not been previously implicated in ALS, consistent evidence of dysregulated cholesterol metabolism—particularly reduced HDL and apoA-I levels—in ALS and related neurodegenerative disorders supports the biological plausibility of its protective role identified in this study.^25^^,^^26^

Together, these lines of evidence indicate that ABCA1 may represent a critical molecular link between lipid metabolic imbalance and ALS pathogenesis. However, its causal role, diagnostic potential, and temporal-spatial expression patterns in ALS remain unexplored, motivating the present multiscale investigation.

## Results

### Overview of the multiscale systems analysis pipeline

**Figure 1:**
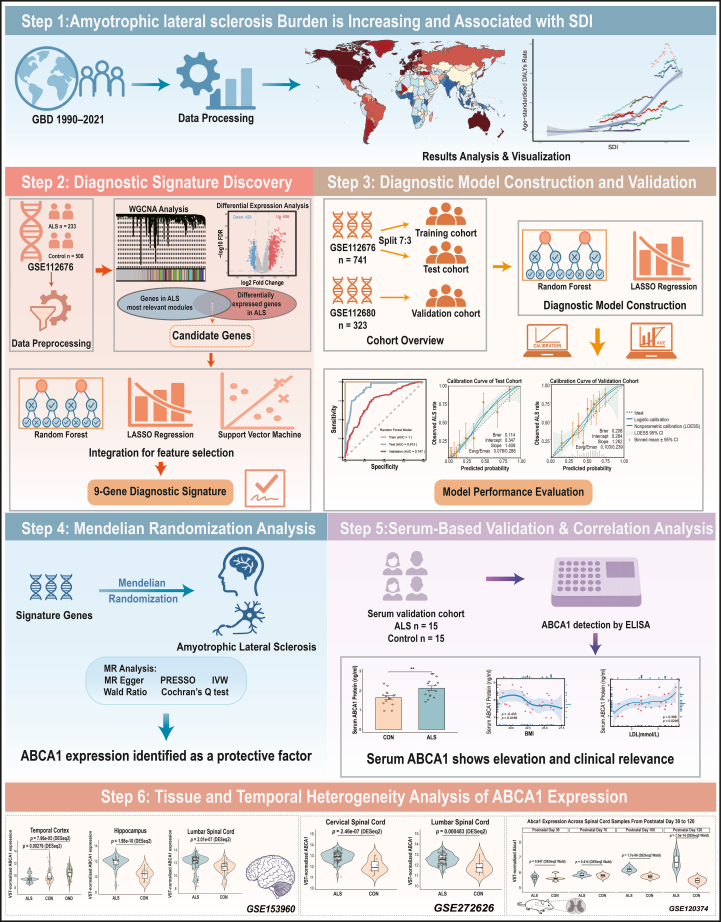
Schematic overview of the multiscale systems analysis pipeline for ALS biomarker discovery The workflow integrates global epidemiological analyses, transcriptomic profiling with machine learning-based signature construction and external validation, Mendelian randomization, and serum-level quantification with clinical correlations, together with surveys of ABCA1 expression across tissues and over time using public datasets. Across these analytic layers, ABCA1 consistently emerged as a leading candidate with evidence consistent with a protective association with ALS.

We established a multiscale analytical framework integrating epidemiological, transcriptomic, genetic, and clinical dimensions to systematically identify and validate potential biomarkers for ALS (Figure 1). The pipeline encompassed large-scale burden assessment, diagnostic signature discovery, machine-learning-based model construction and validation, Mendelian randomization for causal inference, and serum-level quantification with clinical correlations, complemented by the spatial-temporal profiling of *ABCA1* expression. This integrative strategy enabled the cross-validation of findings across population-, molecular-, and individual-level datasets, providing convergent evidence supporting ABCA1 as a candidate protective factor in ALS.

### The global motor neuron diseases burden has increased over time and is strongly correlated with the socio-demographic index

To contextualize the growing need for early diagnostic biomarkers in ALS, we first examined the global burden of MNDs via the GBD dataset (1990–2021), which provides age-standardized incidence, prevalence, mortality, and DALYs across 204 countries and territories. Although MNDs constitute multiple disease entities, ALS constitutes the predominant subtype and is therefore the primary focus of our downstream transcriptomic analyses and targeted serum protein validation.^2^

**Figure 2:**
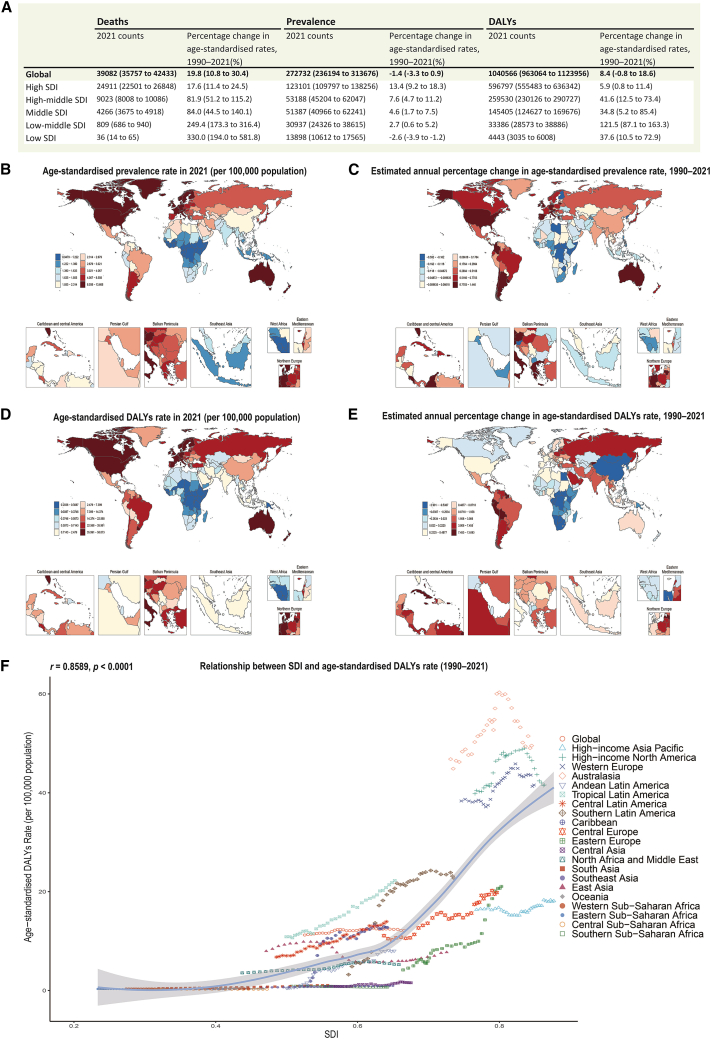
Global trends and socio-demographic patterns in the burden of motor neuron diseases (MNDs), 1990–2021 (A) Summary table lists the total number of MND-related deaths, prevalence cases, and DALYs in 1990 and 2021, along with the percentage changes in age-standardized rates across five Socio-demographic Index (SDI) strata. (B–C) Global maps show (B) age-standardized prevalence rates of MNDs in 2021, and (C) estimated annual percentage change (EAPC) in prevalence from 1990 to 2021. (D–E) Global maps show (D) age-standardized DALYs rates in 2021, and (E) EAPC in DALYs over the same period. (F) Scatterplot of age-standardized DALYs rates in 2021 versus the national SDI, with each point representing a country and colored by GBD superregion. A LOESS smoother (nonparametric) is overlaid with a 95% pointwise confidence band (shaded). The correlation coefficient (Spearman’s *r*) and *p* value are indicated. Notes: EAPC is estimated from a log-linear regression of ASR over 1990–2021. EAPC >0 indicates the corresponding rate increased from 1990 to 2021; EAPC <0 indicates it decreased.

In 2021, high-SDI regions, including North America, Western Europe, and Australasia, exhibited the highest age-standardized rates of MND incidence, prevalence, mortality, and DALYs, with prevalence exceeding 8 cases per 100,000 population in several countries (Figures 2A, 2B, and 2D; Table S1; Figures S1A–S1D). However, temporal trends in disease burden varied substantially across regions. On the basis of the estimated annual percentage change, DALYs increased most rapidly in low- and middle-SDI regions, such as Eastern Europe and Sub-Saharan Africa (Figure 2E), while the most marked increases in prevalence occurred in high-SDI countries, such as the United States, Australia, and several Western European nations (Figure 2C). The incidence rates rose most prominently in Eastern Europe, Russia, and South America, suggesting differential drivers of MNDs burden across regions and metrics (Figures S1B and S1D).

We further analyzed age- and sex-specific patterns of MNDs burden via GBD stratified data (Figures S1E–S1H). Across all four metrics (incidence, prevalence, mortality, and DALYs), the burden of MNDs increased steadily with age, reaching its peak in the seventh to eighth decades of life. Moreover, males consistently exhibited a greater disease burden than females across all age groups, particularly in terms of incidence and DALYs.

Crucially, we identified a robust positive association between a country’s SDI and its 1990–2021 age-standardized DALYs rate (Spearman’s *r* = 0.8589, *p* < 0.0001; Figure 2F), indicating that more socioeconomically developed nations experience a disproportionately higher MNDs burden. These findings confirm the escalating global impact of MNDs and highlight the urgent need to identify sensitive biomarkers and therapeutic targets, particularly for ALS, which accounts for approximately 80–90% of MND cases,^1^^,^^2^ to facilitate early detection and intervention in both high-burden and rapidly emerging regions.

### Weighted gene co-expression network analysis identifies amyotrophic lateral sclerosis-associated gene modules

**Figure 3:**
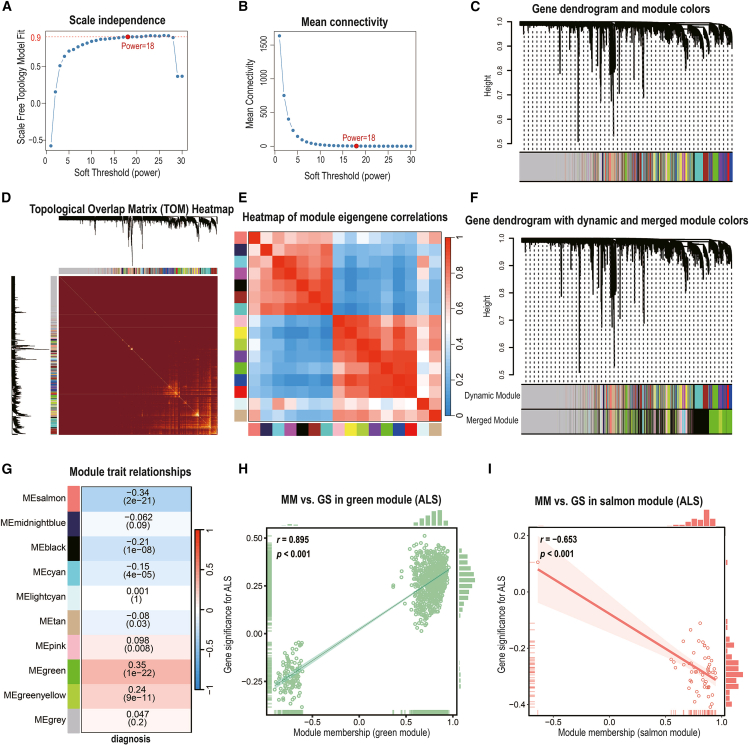
Weighted gene co-expression network analysis identifies ALS-associated modules in whole-blood transcriptomes (A–B) Determination of the soft-thresholding power (β = 18) for scale-free topology via scale independence (A) and mean connectivity (B) plots. (C) Gene dendrogram shows initial module detection via hierarchical clustering of topological overlap. (D) Topological overlap matrix heatmap, with brighter colors indicating stronger gene-gene co-expression. (E) Heatmap of module eigengene correlations across all detected modules. (F) Refined gene dendrogram after merging similar modules via eigengene similarity (merge cut-off height = 0.15). (G) Module-trait correlation heatmap shows associations between module eigengenes and ALS diagnosis. The color intensity represents the correlation between module eigengenes and ALS diagnosis. The numbers within the heatmap indicate Spearman’s r and the corresponding *p* values in parentheses. (H–I) Scatterplots of gene significance (GS) versus module membership (MM) in the green (H) and *salmon* (I) modules. Pearson correlation coefficients (*r*) are shown for each module.

To elucidate the coordinated gene expression patterns associated with ALS, we performed WGCNA on whole-blood transcriptomes from the GSE112676 cohort. Initial clustering identified 16 co-expression modules, which were subsequently merged into 9 consensus modules on the basis of eigengene correlations exceeding 0.85 (merge cut-off height = 0.15), excluding the unassigned *gray* module (Figures 3A–3F).

Module‒trait correlation analysis revealed two modules most strongly associated with ALS status: the *green* module showed a robust positive correlation (Pearson’s *r* = 0.35, *p* = 1.1 × 10^−22^), while the *salmon* module demonstrated a significant negative correlation (Pearson’s *r* = −0.34, *p* = 2.2 × 10^−21^) (Figure 3G). These associations indicated that the *green* module genes were collectively upregulated in ALS, whereas the *salmon* module genes were downregulated. Consistent with these findings, module membership (MM) values were strongly correlated with gene significance (GS) for ALS within both modules, confirming that genes most central to each module’s network structure were also those most strongly associated with disease status (*green*: Pearson’s *r* = 0.895, *p* < 0.001; *salmon*: Pearson’s *r* = −0.653, *p* < 0.001; Figures 3H and 3I; Table S3).

Functional enrichment analysis revealed that the *green* module was significantly enriched for genes involved in RNA splicing, the oxidative stress response, protein ubiquitination, and nuclear organization, as well as immune-related pathways, including NOD-like receptor signaling and autophagy (all FDRs <0.05; Figure S2C; Table S6). Notably, these biological processes are consistent with mechanisms previously implicated in ALS, such as disrupted RNA metabolism, impaired stress responses, and altered immune signaling.^4^^,^^27^

Collectively, WGCNA revealed two biologically coherent gene networks: a *green* module upregulated in ALS, enriched for RNA splicing, oxidative stress response, and immune-related processes, and a *salmon* module downregulated in ALS without significant functional enrichment. These transcriptional modules provide a robust framework for prioritizing hub genes and advancing integrative biomarker discovery in subsequent analyses.

### Differentially expressed genes and their overlap with amyotrophic lateral sclerosis-associated modules

**Figure 4:**
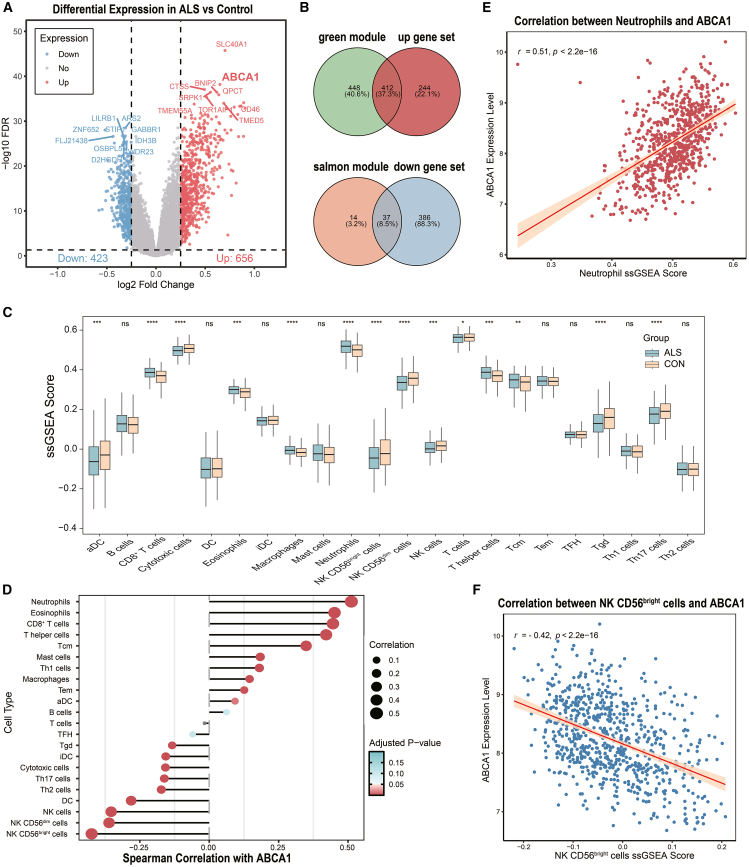
Differential gene expression, module overlap, and immuno-transcriptomic associations with *ABCA1* in ALS (A) Volcano plot of differentially expressed genes (DEGs) in ALS versus control samples. A total of 656 upregulated and 423 downregulated genes were identified (FDR <0.05, |log_2_ Fold Change| > 0.25). The top 10 upregulated and top 10 downregulated genes, ranked by the false discovery rate (FDR), are labeled. (B) Venn diagrams showing the overlap between the DEGs and ALS-related WGCNA modules. The *green* module overlaps with upregulated genes, and the *salmon* module overlaps with downregulated genes. (C) Boxplots of ssGSEA scores for 22 immune cell types in patients with ALS versus controls. Significant differences were assessed via the Wilcoxon test. (D) Correlation matrix of Spearman’s correlation coefficients between *ABCA1* expression and ssGSEA immune cell scores. (E–F) Scatterplots show correlations between *ABCA1* expression and the neutrophil score (E) and the NK CD56^bright^ cell score (F), with 95% confidence intervals shown as shaded bands. Asterisks indicate significance levels as follows: ns, not significant; ∗*p* < 0.05, ∗∗*p* < 0.01, ∗∗∗*p* < 0.001, ∗∗∗∗*p* < 0.0001.

To further characterize transcriptional alterations in the blood of patients with ALS, we performed differential expression analysis between patients with ALS and healthy controls. With thresholds of FDR <0.05, and |log_2_ FC| > 0.25, a total of 1,079 significant DEGs were identified, comprising 656 upregulated and 423 downregulated genes (Figure 4A; Table S4).

Among the most upregulated genes in ALS were *SLC40A1*, *ABCA1*, *CTSS*, *SRPK1*, *BNIP2*, and *QPCT*, whereas the top downregulated genes included *ARS2*, *ZNF652*, *STIP1*, and *GABBR1*. Notably, several key upregulated genes (e.g., *SLC40A1*, *ABCA1*, *BNIP2*, and *QPCT*) overlapped with hub genes from the ALS-associated *green* module identified by WGCNA, reinforcing their biological relevance (Table S3). The heatmap of the top 50 DEGs (Figure S2A) revealed clear separation between the ALS and control samples, supporting the robustness of the observed expression changes.

There was a notable overlap between the DEGs and the ALS-associated modules identified by WGCNA. Specifically, 412 of the 656 upregulated DEGs belonged to the *green* module, whereas 37 of the 423 downregulated DEGs were included in the *salmon* module (Figure 4B). The substantial overlap indicates that these two modules captured a significant portion of ALS-related transcriptional alterations and underscores their potential relevance as candidate gene sets for subsequent machine learning-based diagnostic analyses.

Functional enrichment analysis revealed substantial biological coherence among the upregulated DEGs, with significant enrichment observed in pathways related to RNA metabolism, particularly RNA splicing, spliceosome-mediated RNA processing, and regulation of RNA processing. Other prominent pathways included responses to oxidative stress and protein ubiquitination (Figure S2B). These findings mirrored the functional characteristics identified within the *green* module. The downregulated DEGs were also significantly enriched in several biological pathways (Table S6).

Additionally, ssGSEA revealed distinct immune cell infiltration patterns between patients with ALS and controls (Figure 4C). ALS samples presented significantly elevated enrichment scores for macrophages and neutrophils, whereas several T cell subsets and NK cells presented decreased enrichment scores, indicating a complex pattern of systemic immune dysregulation in ALS.

Collectively, these findings delineate a distinct transcriptomic landscape in ALS blood, marked by the upregulation of immune- and stress-responsive genes and accompanied by peripheral immune remodeling. Integrating DEGs with ALS-associated WGCNA modules yielded a refined panel of 449 candidate genes, which served as the input for downstream machine learning-based biomarker discovery.

### Machine learning defines a cross-validated nine-gene signature for amyotrophic lateral sclerosis diagnosis

**Figure 5:**
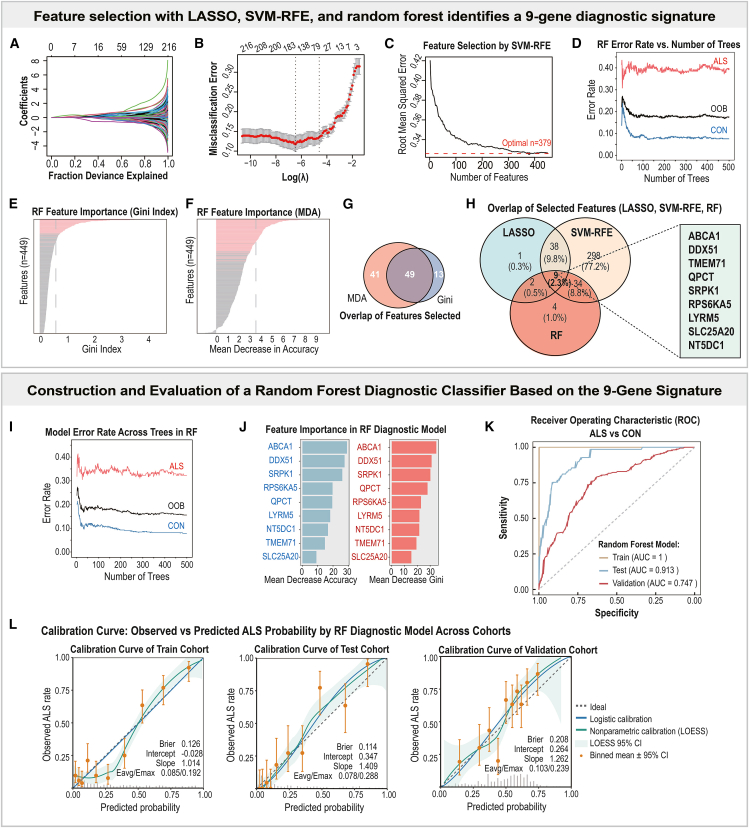
Identification and validation of a nine-gene diagnostic signature for ALS using machine learning approaches (A–B) LASSO feature selection: (A) coefficient trajectories along the regularization path; (B) 10-fold cross-validation curve for λ selection (λ.1se used to determine the optimal feature subset). (C) SVM-RFE recursive feature elimination showing the root mean squared error (RMSE) across different feature counts. (D–F) RF feature selection and performance assessment: (D) feature importance ranked by the Gini index; (E) feature importance ranked by the mean decrease in accuracy (MDA); (F) error-rate curves for ALS, control, and out-of-bag (OOB) samples across increasing tree numbers. In (D–E), pink lines indicate the top 20% ranked genes (*p* < 0.05), while gray bars represent genes not retained in the final feature set. (G) Venn diagram shows the overlap between the MDA- and Gini-based feature sets within the RF model. (H) Intersection of selected features across the three algorithms (LASSO, SVM-RFE, RF), yielding a nine-gene diagnostic signature: *ABCA1, DDX51, TMEM71, QPCT, SRPK1, RPS6KA5, LYRM5, SLC25A20*, and *NT5DC1*. (I–J) Construction of the RF diagnostic classifier based on the nine-gene signature: (I) model error rates across trees for ALS, control, and OOB groups; (J) variable importance within the final RF model based on both Gini and MDA criteria. (K) Receiver operating characteristic (ROC) curves demonstrate the discriminative performance of the RF classifier in the training, internal testing, and external validation cohorts. (L) Calibration curves showing agreement between predicted and observed ALS probabilities across the three cohorts, including ideal, logistic, and nonparametric (LOESS) calibration lines with 95% confidence intervals.

To develop a robust diagnostic panel from blood transcriptomic data, we applied three complementary machine-learning algorithms (LASSO logistic regression, RF, and SVM-RFE) to the 449 candidate genes derived from the intersection of DEGs and ALS-associated WGCNA modules (Figures 5A–5H). Features selection using LASSO regression (Figures 5A and 5B) retained 50 genes with nonzero coefficients. SVM-RFE yielded a broader set of 379 genes with the lowest root mean squared error (RMSE; Figure 5C). RF identified 49 top-ranked predictors on the basis of both the Gini index and the mean decrease in accuracy (Figures 5D–5G). The intersection of all three algorithms yielded a nine-gene consensus signature (Figure 5H; Table S7): *ABCA1*, *DDX51*, *LYRM5*, *NT5DC1*, *QPCT*, *RPS6KA5*, *SLC25A20*, *SRPK1*, and *TMEM71*.

To evaluate diagnostic performance, two classifiers were constructed based on this nine-gene panel. The RF model demonstrated excellent discrimination, with area under the ROC curve (AUC) values of 1.00, 0.91, and 0.75 for the training, internal test, and external validation cohorts, respectively (Figure 5K). The LASSO model also achieved consistent yet moderate performance (AUC = 0.86, 0.89, and 0.72; Figure S3C), suggesting partial generalizability across cohorts.

Calibration curve analysis was conducted to further evaluate the reliability and calibration performance of the diagnostic models.

For the RF classifier (Figure 5L), the predicted probabilities showed strong agreement with the observed ALS rates across cohorts. The Brier scores were low (0.126, 0.114, 0.208), intercepts were close to zero (−0.028, 0.347, 0.264), and slopes approached unity (1.014, 1.409, 1.262) in the training, internal test, and external validation sets, respectively. In addition, the average (E_avg_) and maximum (E_max_) calibration errors remained minimal (E_avg_/E_max_ = 0.085/0.192, 0.078/0.288, 0.103/0.239), indicating good overall calibration and reliability of the RF model across datasets.

In contrast, the LASSO classifier (Figure S3D) exhibited higher Brier scores (0.151–0.253) and substantial deviations in slope (1.847–3.125) and intercept parameters, along with larger calibration errors (E_avg_/E_max_ = 0.119/0.341–0.197/0.335). These metrics indicate that the overall calibration performance of the LASSO model was comparatively modest relative to the RF classifier. To explore sex-specific diagnostic heterogeneity, we further conducted sex-stratified analyses of the nine signature genes in the external validation cohort (Figure S4). The single-gene ROC and expression comparisons revealed that *ABCA1* exhibited the strong discriminative power in both sexes—AUC = 0.674 (accuracy = 0.634, sensitivity = 0.583, specificity = 0.696) in males and AUC = 0.688 (accuracy = 0.690, sensitivity = 0.824, specificity = 0.534) in females. Moderate discriminative value was observed for *SRPK1* (AUC = 0.642 in males; 0.659 in females) and *SLC25A20* (AUC = 0.590 in males; 0.732 in females), whereas *LYRM5* and *NT5DC1* showed limited discriminative capacity (AUC <0.53).

These nine genes span multiple ALS-relevant biological domains, including lipid metabolism (*ABCA1*, *SLC25A20*),^19^^,^^28^ mitochondrial function (*LYRM5*),^29^ RNA processing and stress signaling (*DDX51*, *SRPK1*, *RPS6KA5*),^30^^,^^31^^,^^32^ and immune regulation (*QPCT*).^33^ Collectively, these analyses establish a nine-gene diagnostic signature for ALS, in which *ABCA1* and *SRPK1* consistently emerge as the top contributors, exhibiting consistent differential expression across both male and female subgroups.

### ATP-binding cassette transporter A1 expression is causally associated with reduced amyotrophic lateral sclerosis risk

**Figure 6:**
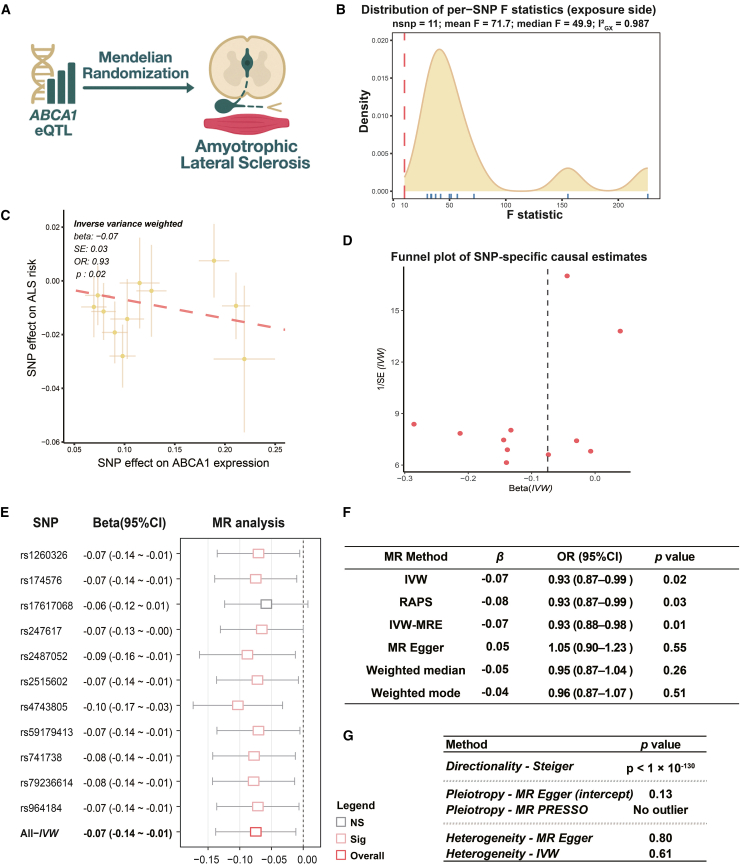
Mendelian randomization (MR) analysis supports a protective causal relationship between *ABCA1* expression and ALS risk (A) Conceptual diagram illustrates the Mendelian randomization (MR) framework used to assess the causal relationship between *ABCA1* expression (eQTL) and ALS. (B) Distribution of F statistics for single nucleotide polymorphisms (SNPs) used as instrumental variables. The red dashed line indicates the conventional threshold (F = 10) for instrument strength, and blue ticks below the axis denote individual SNPs. (C) Scatterplot shows SNP-specific causal estimates, with the red line representing the inverse-variance weighted (IVW) regression slope used to infer the overall causal effect. (D) Funnel plot shows the distribution of SNP-level causal estimates across MR models, used to visually assess potential asymmetry or pleiotropy. (E) Leave-one-out sensitivity analysis, where each SNP was sequentially excluded to evaluate the robustness of causal estimates. Error bars indicate 95% confidence intervals. (F) Summary table of causal effect estimates derived from multiple MR methods, including IVW, RAPS, IVW-MRE, MR-Egger, weighted median, and weighted mode. (G) Summary table of sensitivity analyses, including directionality (Steiger test), pleiotropy (MR-Egger intercept, MR-PRESSO), and heterogeneity (IVW and MR-Egger tests).

To determine whether the diagnostic genes identified through integrative transcriptomic and machine learning analyses exert a causal influence on ALS pathogenesis, we performed two-sample MR using whole-blood *cis*-eQTLs as instrumental variables and ALS GWAS summary statistics as the outcome. Among the nine candidate genes, *ABCA1* emerged as the sole gene showing robust causal evidence across multiple MR methods (Figure 6A).

As shown in Figures 6B and 6C, single-nucleotide polymorphisms (SNPs) associated with *ABCA1* expression (*n* = 11; mean F = 71.7) were used as genetic instruments. Using the inverse-variance weighted (IVW) approach, higher genetically predicted *ABCA1* expression was significantly associated with reduced ALS risk (β = −0.07, OR = 0.93, 95% CI: 0.87–0.99, *p* = 0.02). This association was directionally consistent across RAPS and IVW-MRE methods (*p* = 0.01–0.03), whereas alternative estimators such as the weighted median and MR Egger models produced nonsignificant but directionally similar estimates (Figure 6F).

Forest plot analysis confirmed the stability of these causal estimates across all SNP instruments (Figure 6E). The funnel plot (Figure 6D) showed symmetrical SNP-specific effects, and sensitivity tests indicated no pleiotropy (MR Egger intercept *p* = 0.13; MR-PRESSO global test = no outlier) or heterogeneity (Cochran’s Q: *p* > 0.6; Figure 6G). The Steiger directionality test strongly supported the correct causal direction from *ABCA1* expression to ALS risk (*p* < 1 × 10^−130^).

To ensure the robustness of this finding, replication analyses were performed using three independent ALS GWAS datasets (Figure S5). The European cohort (Nicolas et al.) showed a consistent protective association (β = −0.04, OR = 0.96, *p* = 0.04; Figures S5A–S5E), while the mixed (European + Chinese) and East Asian cohorts demonstrated concordant but nonsignificant trends (β ≈ −0.01 to −0.04, *p* > 0.3; Figures S5F–S5I).

Collectively, these MR analyses provide convergent genetic evidence supporting *ABCA1* as a protective factor against ALS, linking higher *ABCA1* expression to decreased disease susceptibility. Combined with its identification as a WGCNA hub gene, a top DEG, and a major feature in diagnostic models, *ABCA1* consistently emerges as a key ALS-associated biomarker with mechanistic and therapeutic relevance.

### ATP-binding cassette transporter A1 upregulation validated in an in-house serum cohort and its associations with clinical and functional scores

**Figure 7:**
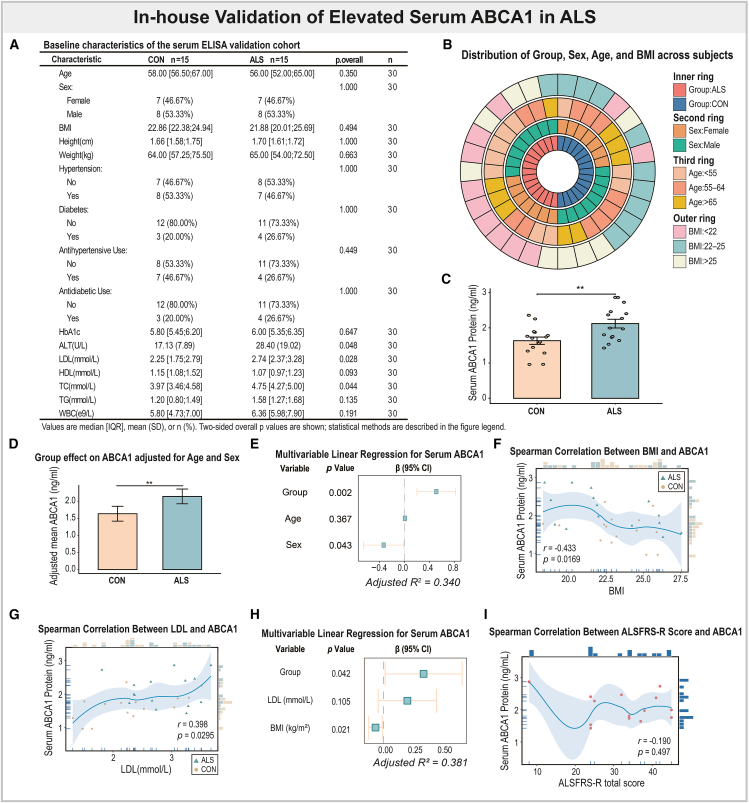
Serum-based validation of ABCA1 elevation and its clinical and functional associations in ALS (A) Baseline characteristics of the in-house serum ELISA validation cohort (*n* = 15 ALS, *n* = 15 controls). (B) Multilayer circular plot shows the distribution of ALS and control subjects by group (inner ring), sex (second ring), age (third ring), and body mass index (BMI, outer ring). (C) Comparison of serum ABCA1 levels between ALS and control groups assessed by Welch’s *t* test. Data are presented as mean ± SEM. (D) Group differences in serum ABCA1 after adjustment for age and sex using linear regression (ABCA1 (ng/ml) ∼ Group + Age + Sex). Error bars represent the 95% confidence interval (CI). (E) Forest plot summarizes multivariable linear regression coefficients for serum ABCA1, with 95% confidence intervals. (F–I) Spearman correlation analyses show associations between serum ABCA1 and (F) BMI, (G) LDL cholesterol, and (I) ALS Functional Rating Scale-Revised (ALSFRS-R) scores. In the correlation plots (F) and (G), circles represent control participants and triangles represent patients with ALS. In panel (I), which analyzes only the ALS cohort (*n* = 15), red markers represent individual patient samples. Marginal histograms and vertical tick marks indicate sample density distributions. Asterisks indicate significance levels as follows: *∗p < 0.05, ∗∗p* < 0.01, ∗∗∗*p* < 0.001.

To further validate the clinical relevance of ABCA1 as a circulating biomarker for ALS, we performed ELISA-based quantification of serum ABCA1 in an independent in-house cohort consisting of 15 patients with ALS and 15 age- and sex-matched healthy controls (Figures 7A, 7B, and S6A). As shown in Figure 7C and Table S10, serum ABCA1 concentrations were significantly higher in ALS compared with controls (*p* = 0.006). Importantly, a multiple linear regression model incorporating storage duration confirmed that sample storage time did not significantly affect ABCA1 levels, thereby excluding pre-analytical bias (Table S10; Figure S6B). After adjustment for age and sex, the group effect remained significant (*p* = 0.002), further supporting the elevation of serum ABCA1 in ALS (Figures 7D and 7E).

We next screened clinical and biochemical variables for association with serum ABCA1 using Spearman correlation across the full panel (Table S12). Three variables met significance: BMI, LDL cholesterol, and body weight. Because BMI and weight are inherently collinear, we retained BMI (as the composite adiposity measure) together with LDL for multivariable modeling. In the correlation analyses, ABCA1 was negatively associated with BMI (Spearman’s *r* = −0.433, *p* = 0.017) and positively associated with LDL (Spearman’s *r* = 0.398, *p* = 0.030; Figures 7F and 7G). In the multiple linear regression including BMI, LDL, and diagnostic group, both BMI and ALS diagnosis remained independent predictors of ABCA1 concentration (*p* = 0.021 and *p* = 0.042, respectively), accounting for 38.1% of variance (adjusted *R*^2^ = 0.381; Figure 7H).

To explore potential clinical and functional implications, we further analyzed the relationships between serum ABCA1 levels and ALS Functional Rating Scale–Revised (ALSFRS-R) total and subdomain scores (Figures 7I and S6C–S6H; Table S13). While neither total nor subscore comparisons reached statistical significance, the overall trends suggested a negative correlation between ABCA1 and ALSFRS-R scores (e.g., total Spearman’s *r* = −0.190, *p* = 0.497; fine motor Spearman’s *r* = −0.199; bulbar Spearman’s *r* = −0.327; gross motor Spearman’s *r* = −0.102; and respiratory Spearman’s *r* = −0.108), implying that higher ABCA1 levels might reflect more advanced functional decline, though these associations did not achieve statistical significance.

Finally, stratified analyses revealed consistent patterns across demographic subgroups (Figures S6I–S6K). Sex-stratified correlations showed broadly similar directions for both males and females, though weaker due to limited sample size, while age-stratified comparisons indicated that the elevation of serum ABCA1 was most apparent in participants aged ≤65 years (*p* = 0.046) but not significant in those >65 years (*p* = 0.052; Table S10).

To gain insight into the potential cellular context of ABCA1 expression in peripheral blood, we examined correlations between ABCA1 mRNA levels and immune cell-type signatures derived from ssGSEA. Notably, ABCA1 expression was positively correlated with the neutrophil score (Spearman’s *r* = 0.51, *p* < 0.001) and negatively correlated with the CD56^bright^ NK cell signature (Spearman’s *r* = −0.42, *p* < 0.001) (Figures 4D–4F). These associations suggest that ABCA1 may be preferentially enriched or functionally coupled with specific innate immune cell populations in the circulation.

Collectively, these findings confirm that serum ABCA1 is robustly elevated in ALS, independent of storage or demographic confounders, and suggest potential links to metabolic state and functional status.

### Spatial and temporal heterogeneity of ATP-binding cassette transporter A1 expression across amyotrophic lateral sclerosis transcriptomic datasets

**Figure 8:**
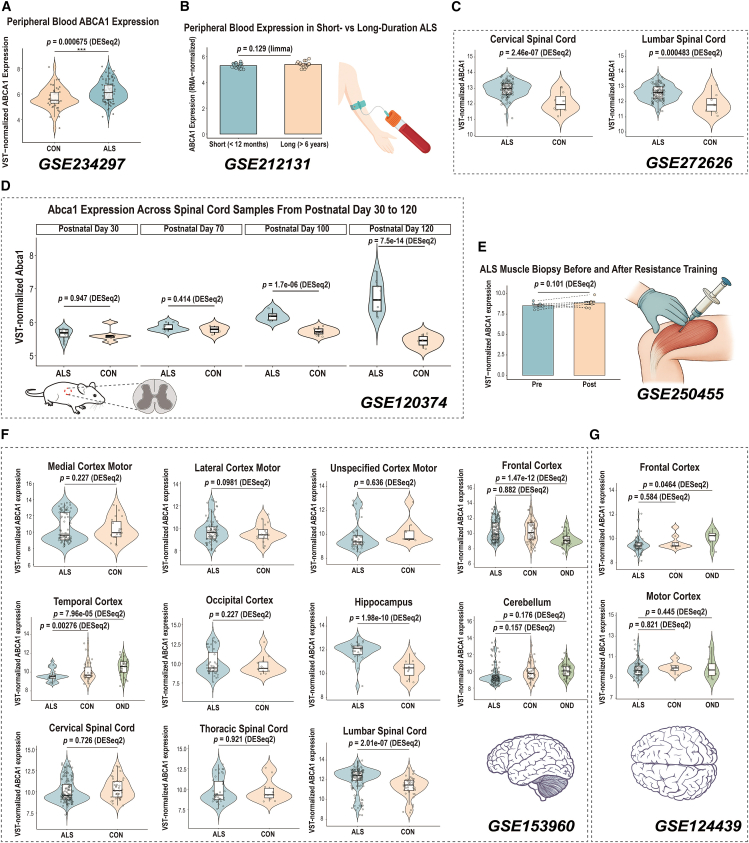
Spatial and temporal heterogeneity of *ABCA1* expression across ALS transcriptomic datasets (A–G) Comparative transcriptomic analyses of *ABCA1* expression across multiple ALS-related datasets. (A) *ABCA1* expression in peripheral blood samples from patients with ALS and healthy controls in the GSE234297 dataset. (B) Differential analysis of ABCA1 expression in peripheral blood samples from patients with ALS with short and long disease durations in the GSE212131 dataset. (C) Comparison of *ABCA1* expression in cervical and lumbar spinal cord postmortem samples from patients with ALS and controls in the GSE272626 dataset. (D) Analysis of *Abca1* expression in spinal cord samples from SOD1-G93A transgenic mice and control mice at four postnatal time points (day 30, day 70, day 100, and day 120) in the GSE120374 dataset. (E) Comparison of *ABCA1* expression in quadriceps muscle biopsy samples collected from patients with ALS before and after resistance training in the GSE250455 dataset. (F) *ABCA1* expression analysis across multiple brain regions, including the cerebral cortex, cerebellum, hippocampus, and spinal cord, in postmortem samples from patients with ALS, healthy controls, and individuals with other neurological disorders (OND) in the GSE153960 dataset. (G) Differential analysis of *ABCA1* expression across cortical subregions in ALS, control, and OND postmortem brain samples in the GSE124439 dataset. Statistical analyses were performed using *DESeq2* or *limma* as indicated in each panel, and *p* values are shown accordingly.

To further delineate the expression landscape of *ABCA1* in ALS, we systematically evaluated its spatial and temporal expression profiles across multiple independent transcriptomic datasets encompassing peripheral blood, spinal cord, skeletal muscle, and brain tissues (Figure 8; Table S14).

In peripheral blood (GSE234297), *ABCA1* expression was significantly upregulated in patients with ALS compared with controls (Log_2_FC = 0.50, *p* = 0.0068; Figures 8A; Table S14). This finding is consistent with our primary transcriptomic analyses and in-house serum validation, both of which demonstrated elevated *ABCA1* levels in ALS, thereby reinforcing its reliability as a reproducible peripheral biomarker across independent cohorts.

In GSE212131, no significant difference was observed between patients with short (<12 months) and long (>6 years) disease duration (Log_2_FC = 0.094, *p* = 0.129; Figures 8B; Table S14).

In spinal cord tissues (Figures 8C and 8F; Table S14), *ABCA1* exhibited region-dependent expression patterns. In the cervical spinal cord, *ABCA1* expression was significantly increased in GSE272626 (log_2_FC = 0.79, *p* = 2.46 × 10^−7^), whereas no significant difference was detected in GSE153960. In the lumbar spinal cord, consistent and significant upregulation was observed across both datasets (GSE272626: log_2_FC = 0.67, *p* = 4.0 × 10^−4^; GSE153960: log_2_FC = 0.82, *p* = 2.01 × 10^−7^). By contrast, *ABCA1* expression in the thoracic spinal cord showed no significant alteration between ALS and control samples. A time-course analysis in SOD1-G93A transgenic mice (GSE120374) revealed a progressive increase in *Abca1* expression across four postnatal stages (day 30, 70, 100, and 120), with corresponding log_2_FC of 0.02, 0.24, 1.24, and 2.39, respectively, and *p* values of 0.95, 0.41, 1.7 × 10^−6^, and 7.5 × 10^−14^ (Figure 8D; Table S14). This pattern indicates a gradual and statistically significant upregulation of *Abca1* from postnatal day 70 onward, reaching its maximum at day 120, consistent with progressive molecular activation during ALS disease progression in the SOD1-G93A model.

In quadriceps muscle biopsy samples (GSE250455), *ABCA1* expression showed a mild but nonsignificant decrease after resistance training (*p* = 0.101; Figure 8E).

In cortical and subcortical tissues, *ABCA1* expression exhibited marked regional heterogeneity across independent postmortem transcriptomic datasets (Figures 8F and 8G; Table S14). In GSE153960, significant *ABCA1* dysregulation was observed in the temporal cortex (log_2_FC = −0.54, *p* = 2.8 × 10^−3^) and hippocampus (log_2_FC = 1.49, *p* = 2.0 × 10^−10^), whereas changes in other cortical regions—including the medial and lateral motor cortices, occipital cortex, and cerebellum—did not reach statistical significance. When comparing ALS with other neurological disorders (OND), *ABCA1* expression in the frontal cortex showed opposite directions across datasets (GSE153960: log_2_FC = 1.52, *p* = 1.5 × 10^−12^; GSE124439: log_2_FC = −0.54, *p* = 0.046). In the temporal cortex, *ABCA1* was significantly downregulated in GSE153960 (log_2_FC = −0.95, *p* = 8.0 × 10^−5^), with no corresponding comparison available in GSE124439.

Together, these analyses demonstrate that *ABCA1* expression in ALS displays marked spatial and temporal heterogeneity, characterized by consistent elevation in peripheral blood and spinal cord, particularly within the lumbar segment, as well as dynamic modulation across cortical and hippocampal regions. These findings indicate that *ABCA1* dysregulation in ALS may reflect the interplay between systemic metabolic alterations and region-specific molecular processes that accompany disease progression.

## Discussion

We present an integrative, multiscale analytical framework that connects global epidemiological trends, blood transcriptomics, machine learning-based diagnostic modeling, genetic causal inference, and serum-level validation to systematically investigate biomarkers for ALS. Using GBD data, we confirmed a steadily increasing global ALS burden, particularly pronounced in high-SDI regions, underscoring the urgent need for novel diagnostic approaches and therapeutic targets. Systems-level transcriptomic analysis identified two ALS-associated co-expression modules and 1,079 differentially expressed genes, from which a nine-gene diagnostic signature was derived through orthogonal feature-selection algorithms. Among these, *ABCA1*, *SLC25A20*, and *SRPK1* emerged as the top contributors. Mendelian randomization analysis demonstrated that genetically elevated *ABCA1* expression is causally associated with a reduced risk of ALS, highlighting its potential protective role. At the protein level, serum validation further confirmed significantly increased ABCA1 concentrations in patients with ALS, associated with lower BMI and higher LDL levels. Moreover, cross-tissue and temporal analyses revealed region-dependent expression patterns of *ABCA1*, with consistent elevation in peripheral blood and lumbar spinal cord and a progressive upregulation during disease advancement in the SOD1-G93A mouse model.

The global burden of ALS continues to rise, as confirmed by age-standardized trends in incidence, prevalence, mortality, and DALYs over the past three decades. High-SDI regions—such as North America, Western Europe, and Australasia—consistently present the greatest absolute burden, likely reflecting a combination of older populations, advanced diagnostic infrastructure, and improved survival with supportive care. However, the most rapid percentage increases in mortality and DALYs were observed in low- and middle-SDI countries, indicating an expanding and uneven distribution of the ALS burden worldwide (Figures 2 and S1). These trends may reflect, in part, improved the recognition of ALS in previously underdiagnosed settings, but they also raise concerns about persistent disparities in care delivery, delayed diagnosis, and lack of access to disease-modifying treatments. Environmental exposure, nutritional deficiencies, and comorbidities may further compound vulnerability in these regions.

This epidemiological divergence underscores the urgent need for scalable, accessible biomarkers that can facilitate early diagnosis and risk stratification of ALS, particularly in resource-limited environments. Current diagnostic paradigms rely heavily on clinical and electrophysiological criteria that become definitive only in later stages of disease. The absence of reliable, blood-based biomarkers hampers early detection, delays therapeutic intervention, and contributes to global inequity in ALS outcomes.

To address this diagnostic gap and elucidate the molecular mechanisms underlying ALS, we next performed systems-level transcriptomic analyses to identify disease-associated expression patterns in peripheral blood. Differential expression and WGCNA revealed biologically coherent modules strongly correlated with ALS status, particularly those enriched in RNA processing, oxidative stress, and immune-related pathways. By integrating these findings with machine learning-based feature selection, we derived a nine-gene diagnostic signature that demonstrated robust discriminative performance across both internal and external validation cohorts. Among these nine genes, *ABCA1*, *SLC25A20*, and *SRPK1* consistently ranked as the top contributors, converging on pathways involved in lipid metabolism, mitochondrial activity, and stress-response signaling, which are highly relevant to the pathophysiology of ALS.

Building upon these molecular and diagnostic insights, we next applied MR to determine whether any of the identified genes exerted a causal effect on ALS susceptibility. Interestingly, despite the protective association observed in our MR analysis, *ABCA1* expression was consistently elevated across patients with ALS, as evidenced by external transcriptomic datasets, serum ELISA validation, and external cross-tissue analyses. This observation initially appears paradoxical—if genetically higher *ABCA1* expression reduces ALS risk, why is it increased in affected individuals?

To clarify this discrepancy, we carefully examined the ancestry composition of the datasets used in both the MR and transcriptomic analyses. All primary GWAS sources applied in the MR analysis, including those by van Rheenen et al. and Nicolas et al., as well as our discovery and validation transcriptomic cohorts, were derived from European populations,^34^^,^^35^ thereby minimizing potential population stratification bias. The MR analyses based on these European datasets consistently demonstrated a protective causal association between higher genetically predicted *ABCA1* expression and reduced ALS risk (Figures 6 and S4; Table S8). Furthermore, replication analyses using Asian or mixed-ancestry GWAS data (Benyamin et al.; Iacoangeli et al.) did not reach statistical significance but showed concordant protective directions (Figure S5; Table S8), reinforcing the cross-population robustness of this causal inference. Given this evidence, population stratification can be reasonably excluded as the source of the observed discrepancy. Moreover, our independently collected Chinese serum cohort also revealed significantly elevated ABCA1 protein levels, further confirming that this upregulation is reproducible across distinct ethnic backgrounds and biological levels.

Having ruled out population structure effects, we therefore interpret the elevated ABCA1 levels as a compensatory upregulation serving a potentially protective role rather than a pathogenic trigger. This interpretation is supported by both animal and human data. In the SOD1-G93A mouse model, *ABCA1* expression increased significantly along the disease course (Figure 8), indicating progressive activation concurrent with disease advancement. Consistently, in our in-house serum cohort, ABCA1 levels exhibited a negative trend with ALSFRS-R total and subdomain scores (Figures 7I and S6C–S6H), suggesting that patients with greater functional impairment tended to show higher ABCA1 concentrations, although these associations did not reach statistical significance and warrant further validation in larger populations. *ABCA1* functions as an inducible cholesterol efflux transporter and immunomodulator,^17^^,^^18^^,^^19^ that is widely expressed in peripheral immune cells (e.g., monocytes and macrophages) and in the CNS (astrocytes and microglia), where it responds to sterol and inflammatory signals.^20^^,^^21^^,^^22^ Notably, previous studies have shown that *ABCA1* can mediate anti-inflammatory responses; specifically, its interaction with apoA-I in cholesterol-enriched macrophages activates the JAK2/STAT3 signaling cascade, thereby attenuating proinflammatory cytokine production.^21^ Furthermore, ABCA1 induction itself has been implicated as a downstream effector of anti-inflammatory pathways, such as LXR activation, which promote the resolution of inflammatory lesions.^22^ Given that ALS is characterized by systemic inflammation and metabolic dysregulation, it is plausible that ABCA1 upregulation represents a compensatory mechanism to counteract ongoing inflammatory and metabolic stress.

This compensatory framework reconciles the apparent discrepancy between the genetically inferred protective effect and the elevated expression observed in patients: genetically higher baseline *ABCA1* expression may reduce disease susceptibility, whereas its further induction during disease progression likely reflects an adaptive effort to restore lipid-immune homeostasis. Future longitudinal and functional studies will be necessary to determine whether elevated ABCA1 levels are associated with slower disease progression or distinct metabolic phenotypes.

The proposed neuroprotective role of ABCA1 in ALS is also consistent with findings from other neurodegenerative disorders. In Alzheimer’s disease, ABCA1 has been identified as a genetic risk factor; its deficiency promotes Aβ accumulation and cognitive decline, whereas its overexpression reduces amyloid pathology.^36^^,^^37^^,^^38^^,^^39^ In Parkinson’s disease, ABCA1 is thought to modulate glial inflammation and lipid homeostasis, although evidence remains more limited.^22^ More broadly, ABCA1 has been implicated in maintaining blood-brain barrier integrity, attenuating neuroinflammation, and supporting synaptic function across models of AD, PD, and stroke.^22^ Notably, the pharmacologic activation of ABCA1 through LXR agonists has shown therapeutic benefits in several of these conditions.^22^^,^^37^^,^^38^^,^^39^ Collectively, these findings identify ABCA1 as a key regulator of lipid and immune homeostasis in neurodegeneration, potentially extending its relevance to ALS. The correlations we observed between ABCA1 levels and patients’ metabolic parameters provide additional biological context. Within the ALS group, serum ABCA1 levels were positively correlated with LDL cholesterol and inversely correlated with BMI (Figures 7F–7I), suggesting a potential link between ABCA1 expression and lipid metabolic status. These relationships are intuitive in light of known metabolic alterations in ALS. Notably, multiple linear regression analysis confirmed that the inverse association between BMI and serum ABCA1 levels remained significant after adjusting for both ALS diagnosis and LDL cholesterol levels, indicating an independent and robust relationship.

Patients with ALS often develop hypermetabolism and dyslipidemia, and intriguingly, those with higher lipid levels tend to fare better clinically.^40^^,^^41^ For example, a classic study by Dupuis et al. revealed that the frequency of hyperlipidemia (elevated LDL or total cholesterol) is significantly higher in patients with ALS than in healthy controls and that patients with ALS with an abnormally high LDL/HDL ratio survive, on average, more than one year longer than those with lower ratios.^41^ However, Mendelian randomization analyses by Zeng and Zhou and by Wang et al. have demonstrated that genetically elevated LDL cholesterol is causally associated with an increased risk of ALS, indicating that LDL acts as a risk factor rather than a protective one. This apparent discrepancy suggests that the elevated LDL levels observed in patients likely reflect a secondary metabolic response to disease progression, rather than a beneficial effect of LDL itself.^42^^,^^43^ In contrast, low BMI and rapid weight loss have been consistently associated with increased ALS risk and poorer prognosis, whereas individuals maintaining higher or stable body weight generally experience slower disease progression.^44^^,^^45^^,^^46^

Overall, the causal relationships among ABCA1, LDL cholesterol, and BMI appear to be largely independent. Mendelian randomization studies have established LDL as a genetic risk factor for ALS, whereas higher genetically predicted BMI exerts a protective effect against disease susceptibility. In contrast, elevated genetically predicted ABCA1 expression confers a reduced ALS risk. Therefore, the correlations observed between ABCA1, LDL, and BMI in our cohort are unlikely to represent shared causal mechanisms. Instead, they likely reflect the intrinsic biological functions of ABCA1 in lipid handling and metabolic regulation. As a pivotal cholesterol efflux transporter, ABCA1 upregulation may occur as an adaptive response to metabolic stress, facilitating lipid redistribution and maintaining systemic homeostasis. This interpretation supports a model in which ABCA1 acts independently as a compensatory regulator that buffers metabolic and inflammatory stress during ALS progression, rather than mediating effects through LDL or BMI pathways.

In conclusion, our study identifies ABCA1 as a promising therapeutic candidate for ALS. Furthermore, we systematically characterized the spatiotemporal heterogeneity of ABCA1 expression across multiple tissues in both patients with ALS and disease models. Collectively, these findings offer fresh insights into the link between systemic metabolic adaptation and neurodegeneration, providing a rationale for further exploration of ABCA1-targeted interventions.

### Limitations of the study

This study has several limitations. First, our transcriptomic analyses were conducted in peripheral blood, which, while accessible, may not fully capture disease-relevant molecular changes occurring in the central nervous system. Second, although our findings support a protective role for *ABCA1*, the specific molecular mechanisms underlying this relationship remain to be elucidated through further experimental studies, particularly to clarify whether its effects are primarily mediated by lipid metabolism, immune regulation, or their interplay. Third, the diagnostic model’s performance declined in the external validation cohort, likely due to intrinsic batch effects across cohorts and sequencing platforms. Fourth, for the MR analysis, sex-stratified eQTL/GWAS summary statistics were not available; consequently, we could not directly test whether the inferred protective association for ABCA1 is consistent between men and women.

These limitations highlight the need for future research focusing on how ABCA1 exerts its protective effects within the central nervous system. Future studies should employ cellular and animal models to elucidate the underlying neuroprotective mechanisms and, in particular, to investigate the interplay between peripheral ABCA1 expression and its central actions in maintaining lipid and immune homeostasis during ALS progression. In this context, our ssGSEA-based immune deconvolution analysis revealed that ABCA1 expression was most strongly associated with neutrophil and NK CD56^bright^ cell signatures. Notably, recent cohort evidence by Gong et al. identified NK CD16^−^ CD56^bright^ cells as a protective subset associated with slower ALS progression and better prognosis.^47^ Building on these findings, future mechanistic studies should explore the functional relationship between ABCA1 and this NK cell population, as well as their potential cross-talk between the peripheral immune system and the central nervous system.

## Resource availability

### Lead contact

Further information and requests for resources, data, and materials should be directed to and will be fulfilled by the Lead Contact, Yuming Xu (email: xuyuming@zzu.edu.cn).

### Materials availability

This study did not generate new unique reagents, materials, or cell lines. Human serum samples used in this study were collected under institutional ethical approval (Ethics Approval No. 2022-KY-0386-002). The corresponding serum data have been de-identified, and necessary anonymized data have been made publicly available to ensure transparency while maintaining participant confidentiality.

### Data and code availability

• All transcriptomic datasets analyzed in this study are publicly available from the NCBI GEO. Accession numbers are listed in the key resources table. GBD data were obtained from the Global Health Data Exchange portal (https://vizhub.healthdata.org/gbd-results/). Summary-level GWAS data and eQTL data for ABCA1 were obtained from the IEU OpenGWAS Project (https://gwas.mrcieu.ac.uk/). The processed GBD source data and other derived data supporting the findings of this study are available in the supplemental information (Tables S2, S9, and S13).
• All analyses and visualization codes used in this study, including GBD data processing, transcriptomic analyses, machine learning, and Mendelian randomization, have been deposited at GitHub and are publicly available as of the date of publication. The link is listed in the software and algorithm section of the key resources table.
• Any additional information required to reanalyze the data reported in this article is available from the lead contact upon request.

## Acknowledgments

The authors thank all participants who voluntarily enrolled in the ALS validation cohort used in this study. We also acknowledge and appreciate the contributions of the original data generators for the publicly available GEO transcriptomic datasets, as well as the IEU OpenGWAS project and the original GWAS consortia for providing summary statistics. Finally, we are grateful to the four anonymous reviewers for their valuable comments and suggestions, which significantly helped improve the quality of this article.

The authors’ work was supported by the key scientific and technological breakthrough project in Henan province (Grant 232102311229), the joint construction project of Henan Province medical science and technology key research program (Grant LHGJ20210293) and the 10.13039/501100002858China Postdoctoral Science Foundation (Grant 2022 M722875 and 2023 T160600).

## Author contributions

Q.L. and G.Z. contributed equally to this work. Q.L. and G.Z. conceived and designed the study, performed the data analysis, and wrote the article. Y.C.Z. and H.Z.H. were responsible for sample collection, processing, and clinical data acquisition. T.Z. and H.L.Z. contributed to data visualization and figure preparation. H.L. and Y.X. supervised the project, provided critical revisions, and served as corresponding authors. All the authors reviewed and approved the final article.

## Declaration of interests

The authors declare no competing interests.

## STAR★Methods

### Key resources table

**Table:** 

REAGENT or RESOURCE	SOURCE	IDENTIFIER
**Biological samples**		

Human serum samples from ALS patients and controls	In-house cohort, Zhengzhou University	Ethics Approval No. 2022-KY-0386-002

**Critical commercial assays**		

Human ABCA1 ELISA Kit	FineTest (Wuhan, China)	Cat# EH1420

**Deposited data**		

Raw and analyzed data	This paper	Tables S9 and S13
Global Burden of Disease data	Global Health Data Exchange (GHDx)	https://vizhub.healthdata.org/gbd-results/
Gene Expression Omnibus: GSE112676	NCBI GEO	GSE112676
Gene Expression Omnibus: GSE112680	NCBI GEO	GSE112680
Gene Expression Omnibus: GSE120374	NCBI GEO	GSE120374
Gene Expression Omnibus: GSE234297	NCBI GEO	GSE234297
Gene Expression Omnibus: GSE212131	NCBI GEO	GSE212131
Gene Expression Omnibus: GSE272626	NCBI GEO	GSE272626
Gene Expression Omnibus: GSE250455	NCBI GEO	GSE250455
Gene Expression Omnibus: GSE124439	NCBI GEO	GSE124439
Gene Expression Omnibus: GSE153960	NCBI GEO	GSE153960
eQTL data for ABCA1	IEU OpenGWAS Project	https://gwas.mrcieu.ac.uk/; id: eqtl-a-ENSG00000165029
Summary-level GWAS data for ALS	IEU OpenGWAS Project	https://gwas.mrcieu.ac.uk/; id: ebi-a-GCST90027163; ebi-a-GCST005647; ebi-a-GCST004901; ebi-a-GCST90013429

**Software and algorithms**		

R statistical software (v4.3.1)	R Foundation	https://www.r-project.org/
DESeq2	Love et al., 2014^48^	R package (v1.48.2)
limma	Ritchie et al., 2015^49^	R package (v3.64.0)
WGCNA	Langfelder & Horvath, 2008^50^	R package (v1.73)
clusterProfiler	Yu et al., 2012	R package (v3.21)
GSVA	Hänzelmann et al., 2013	R package (v2.20)
caret	Max Kuhn, 2019	R package (v7.0.1)
randomForest	Breiman, 2001^51^	R package (v4.7-1.1)
glmnet	Friedman et al., 2010^52^	R package (v4.1-8)
TwoSampleMR	Hemani et al., 2018^53^	R package (v0.6.15)
pROC	Robin et al.,2011	R package (v1.19.01)
mgcv	Wood, 2017	R package (v1.9-3)
glm	Marschner, 2011	R package (v3.6.2)
ggplot2	Wickham, 2016	R package (v3.5.2)
Code for GBD and ALS Analysis	This paper	https://github.com/DrZoggg/ALS

**Other**		

Microplate reader (for ELISA)	BIO-DL, China	Model: K3 Plus

### Experimental model and study participant details

#### Human subjects

GWAS summary statistics Summary-level data for ALS (outcome) and ABCA1 expression (exposure) were obtained from large-scale GWAS meta-analyses predominantly involving individuals of European ancestry. Detailed cohort information is provided in Table S8. Due to the unavailability of sex-stratified summary statistics in the source datasets, Mendelian randomization analyses were performed on the aggregate population, and sex-specific causal effects could not be assessed. Serum samples were obtained from an in-house retrospective cohort comprising 15 patients with ALS and 15 neurologically healthy controls, all of whom were of Chinese Han ethnicity. ALS diagnosis was established according to the revised El Escorial criteria (Airlie House revision). Control participants were matched for age and sex and had no history of neurodegenerative or metabolic disorders (detailed demographic information is provided in Table S9). All participants provided written informed consent prior to inclusion. The study protocol was approved by the Ethics Committee of Zhengzhou University (Approval No. 2022-KY-0386-002). Blood was collected under fasting conditions, and serum was isolated and stored at −80°C until analysis. We reported differential analyses adjusted for age and sex to control for potential confounding, and performed sex-stratified correlation analyses to evaluate potential differences in ABCA1 expression and its clinical associations between male and female participants, as detailed in the results section.

#### Animal models

Publicly available transcriptomic data from the SOD1-G93A transgenic mouse model of ALS were obtained from the GEO (accession number GSE120374). The dataset includes spinal cord samples from SOD1-G93A and wild-type littermates at postnatal days 30, 70, 100, and 120. All animal procedures for the original study were approved by the respective institutional animal care and use committees, as reported in the source publication.^54^

### Method details

#### Global burden of disease data analysis

To contextualize the epidemiological burden of ALS, we analyzed data on motor neuron diseases (MNDs) from the Global Burden of Disease Study 2021 (https://vizhub.healthdata.org/gbd-results/). Age-standardized incidence rates (ASIRs), prevalence rates (ASPRs), mortality rates (ASMRs), and disability-adjusted life-years (DALYs) were retrieved for 204 countries and territories between 1990 and 2021. Estimates were expressed per 100,000 population, with age standardization on the basis of the GBD reference population.

EAPC was estimated by fitting a log-linear regression model between the natural logarithm of the age-standardized rate (ASR) and calendar year as follows:

ln(ASR)=α+β×(year)+ε

where β represents the annual change in the log-transformed ASR.

#### The EAPC was then calculated as

$$EAPC=100\times \left(e^{\beta }-1\right)$$

Positive EAPC values indicate an increasing trend, while negative values indicate a decreasing trend. Uncertainty intervals for annual ASRs were derived from the GBD-provided 95% uncertainty intervals (UIs), which incorporate sampling and model uncertainty.

The relationship between the regional Socio-demographic Index (SDI) and disease burden was assessed via Spearman correlation.

#### Gene expression datasets and preprocessing

Two publicly available gene expression datasets of peripheral whole blood from patients with ALS were analyzed. The first dataset, GSE112676, contains transcriptome profiles from ALS patients (*n* = 233) and neurologically normal controls (*n* = 508) and was originally published by van Rheenen et al.^55^ The second dataset, GSE112680, includes an independent cohort of ALS patients (*n* = 164) and controls (*n* = 159) profiled on a similar microarray platform. For network construction (WGCNA) and differential expression analysis, the full GSE112676 cohort (*n* = 741) was used without splitting. For diagnostic model training and internal validation, the GSE112676 dataset was randomly divided into a training set (70%) and a test set (30%). Independent external validation was performed with the GSE112680 dataset (*n* = 323).

Raw expression data from the GSE112676 and GSE112680 datasets (Illumina HumanHT-12 v3 and v4 platforms, respectively) were retrieved from the GEO. As raw IDAT files were unavailable, preprocessed expression matrices provided by the original authors, which had undergone background correction, quantile normalization, surrogate variable analysis for batch adjustment, and log_2_ transformation, were used. Probes were mapped to official gene symbols via the Illumina GPL6947 annotation file. Probes with zero expression across all samples were removed. When multiple probes corresponded to the same gene symbol, the probe with the highest average expression was retained. Genes were retained if they had a mapped symbol and Entrez gene ID. The Entrez gene IDs were further mapped to Ensembl gene IDs via the org.Hs.e.g.,.db database. Only protein-coding mRNAs were retained for analysis. Finally, the gene lists from GSE112676 and GSE112680 were intersected, yielding a common set of 18,005 mRNA genes for downstream analyses.

#### Weighted gene co-expression network analysis

Weighted gene co-expression network analysis was performed via the *WGCNA* R package^50^ on the full GSE112676 dataset. An unsigned network was constructed to capture both positively and negatively correlated gene pairs, given that both directions of dysregulation may contribute to disease. The optimal soft-thresholding power (*β*) was selected by evaluating the scale-free topology model fit (R^2^ > 0.9). An adjacency matrix was calculated and transformed into a topological overlap matrix (TOM) to measure network interconnectedness. Genes were clustered via hierarchical clustering on the basis of TOM dissimilarity, and modules were detected via the dynamic tree cut method with a minimum module size of 30 genes. Closely related modules were merged if their eigengene correlation exceeded 0.85 (merge cut-off height = 0.15). Each module eigengene (the first principal component of gene expression within a module) was correlated with the ALS diagnosis status. Modules significantly associated with ALS (*p* < 0.05, Bonferroni-corrected) were initially identified, from which the most strongly positively correlated (*green* module) and most strongly negatively correlated (*salmon* module) were selected for downstream analyses.

#### Differential expression analysis and functional enrichment

Differential gene expression analysis between ALS patients and controls was performed on the full GSE112676 dataset. Linear models were fitted for each gene on the basis of the ALS diagnosis status, and empirical Bayes moderation was applied to improve variance estimation. Differentially expressed genes (DEGs) were defined as those satisfying two criteria: false discovery rate (FDR) < 0.05, and absolute log_2_ fold change (|log_2_FC|) > 0.25.

Gene set enrichment analyses were conducted separately for (i) the full set of DEGs and (ii) genes within the ALS-associated WGCNA modules. Enrichment analyses were performed via the *clusterProfiler* R package. Overrepresentation testing was carried out for Gene Ontology (GO) biological processes and Kyoto Encyclopedia of Genes and Genomes (KEGG) pathways. Pathways were considered significantly enriched if they met the FDR <0.05 thresholds. All enrichment analyses were performed via default parameters unless otherwise specified.^56^

#### Single-sample gene set enrichment analysis

To evaluate immune cell infiltration patterns in ALS peripheral blood samples, we performed single-sample gene set enrichment analysis (ssGSEA) via the *GSVA* package in R. This method computes an enrichment score for each sample and gene set, enabling the quantification of relative immune cell type abundance at the individual-sample level.

A total of 22 immune cell types, including various T cell subsets (e.g., CD8^+^ T cells, Th1 cells, and Tregs), B cells, NK cells, macrophages, dendritic cells, and neutrophils, were analyzed. Cell type–specific marker gene sets were curated from published literature and are provided in Table S5.^57^

ssGSEA scores were calculated on the normalized expression matrix of the ALS transcriptomic dataset (GSE112676). The resulting scores were compared between ALS patients and healthy controls via the Wilcoxon rank-sum test, and Benjamini–Hochberg correction was applied to control for multiple testing.

Correlations between individual immune cell ssGSEA scores and *ABCA1* expression were assessed via Spearman’s rank correlation coefficient, with the results visualized as a heatmap and representative scatterplots.

#### Machine learning for feature selection and diagnostic models

Feature selection was performed using three machine learning algorithms (LASSO logistic regression, RF, and SVM-RFE) on the GSE112676 training set. LASSO logistic regression was conducted via the *glmnet* package.^52^ The regularization parameter (λ) was selected on the basis of the lambda.1se criterion, which retains the smallest number of nonzero coefficients within one standard error of the minimum cross-validation error. Genes with nonzero coefficients at the selected λ were considered as informative features and retained for subsequent model development. Random forest models were built via the *randomForest* package, which specifies 500 trees and balanced class weights. To ensure feature stability, the models were iterated 1,000 times with bootstrapped sampling. The feature importance for each gene was summarized across all iterations by averaging both the mean decrease Gini coefficient and the mean decrease accuracy metrics. Genes ranking within the top 20% by feature importance and meeting the significance threshold (*p* < 0.05) were retained as robust candidates for further analysis. Support vector machine recursive feature elimination was applied via the *caret* package with a linear kernel. Recursive elimination was performed to iteratively exclude the least informative features. The final feature subset was determined by minimizing the cross-validation classification error.

The features selected by the three machine learning methods were intersected, and the genes present in all three algorithms were designated as a nine-gene diagnostic signature for downstream model construction.

To evaluate the diagnostic performance of the nine-gene signature, we constructed a random forest (RF) classifier as the primary model and a LASSO logistic regression model as a comparator. Both models were trained on the GSE112676 training set and internally validated on the held-out test set; final models were then applied to the independent external cohort (GSE112680) to assess generalizability. Discriminative performance was quantified by the area under the receiver operating characteristic (ROC) curve, which was plotted using the *pROC* R package with default parameters.^58^

Model calibration and discrimination were evaluated for both the random forest (RF) and least absolute shrinkage and selection operator (LASSO) diagnostic models across the training, internal testing, and external validation datasets.

Calibration analyses were performed using custom R functions built upon the *pROC*, *ggplot2*, *mgcv*, and *glm* frameworks. For each model–dataset combination, the raw predicted probabilities were compared with the observed ALS status to assess calibration performance.

Predicted probabilities were constrained within [10^−6^,1-10^−6^] to ensure numerical stability. Calibration was evaluated using three complementary approaches.

(1) the ideal 45° reference line, representing perfect calibration;
(2) the logistic calibration line, fitted using

$$\text{logit}\left(y\right)=\alpha +\beta \cdot \text{logit}\left(\hat{p}\right)$$

where the intercept (*α*) and slope (*β*) represent calibration-in-the-large and calibration slope, respectively.

(3) a nonparametric LOESS smoothing curve, with fallback to a generalized additive model (mgcvgam) if local regression failed to converge.

Observed event rates were estimated in 10 quantile-based bins of predicted probability, and 95% Wilson confidence intervals were computed for each bin.

#### Calibration error was quantified using the following metrics

$$E_{avg}=\frac{1}{K}\sum_{k=1}^{K}\left|\bar{y_{k}}-\bar{p_{k}}\right|$$

$$E_{\max}=\max_{k}\left|{\bar{y}}_{k}-{\bar{p}}_{k}\right|$$

where ${\overset{ˉ}{y}}_{k}$ and ${\overset{ˉ}{p}}_{k}$ denote the observed and predicted event probabilities in bin *k*, respectively.

Additional indices included the Brier score, calibration intercept, and calibration slope derived from the logistic regression fit.

#### Mendelian randomization analysis for causal inference

Observational studies cannot fully eliminate confounding factors or establish causality, limiting their ability to identify protective or pathogenic factors in individuals with ALS. To address this, we applied a two-sample MR framework to evaluate whether genetically determined expression levels of the nine hub genes identified from our transcriptomic and machine learning analyses causally influence ALS risk.

For each gene, *cis*-expression quantitative trait loci (*cis*-eQTLs) were retrieved from the GTEx v8 database. Independent SNPs significantly associated with gene expression (*p* < 5 × 10^−8^) were selected, applying clumping to ensure linkage disequilibrium r^2^ < 0.001. ALS outcome data were sourced from the largest available ALS GWAS meta-analysis^34^(van Rheenen et al.; ∼27,205 ALS cases and ∼110,881 controls). The specific GWAS datasets applied are detailed in Table S8. SNP effect sizes (beta coefficients and standard errors) for both the exposure (gene expression) and outcome (ALS risk) were harmonized across datasets.

The inverse-variance weighted (IVW) method served as the primary MR estimator. Sensitivity analyses included MR–Egger regression, weighted median, simple mode, and weighted mode estimators to evaluate robustness. Leave-one-out analysis was used to assess whether the results were driven by any single SNP. Heterogeneity across instrumental variables was quantified using Cochran’s Q statistic, while horizontal pleiotropy was evaluated via the MR–Egger intercept and Mendelian Randomization Pleiotropy RESidual Sum and Outlier (MR-PRESSO) tests. Reverse MR analysis was also conducted to examine potential reverse causation (i.e., whether ALS risk might influence gene expression). All MR analyses were performed via the *TwoSampleMR* R package.^53^

#### Serum ABCA1 protein quantification and clinical correlation

This retrospective cohort study was approved by the Ethics Committee of Zhengzhou University (Approval No. 2022-KY-0386-002). Written informed consent was obtained from all participants.

We enrolled 15 patients diagnosed with ALS according to the revised El Escorial criteria (Airlie House revision) and 15 neurologically healthy controls. Per the revised El Escorial (Airlie House) criteria, ALS diagnosis requires: (1) evidence of lower motor neuron degeneration by clinical, electrophysiological, or neuropathologic examination; (2) evidence of upper motor neuron degeneration by clinical examination; and (3) progressive spread of symptoms or signs within a region and to other regions; together with (4) the absence of electrophysiological/pathological or neuroimaging evidence for other disease processes that could account for the findings.

Peripheral venous blood samples were collected in procoagulant tubes and centrifuged at 1,500 × g for 15 min. The serum supernatant was aliquoted and stored at −80°C until analysis. Serum ABCA1 protein concentrations were quantified via a commercial human ABCA1 ELISA kit (catalog no. EH1420; FineTest, Wuhan, China). The ELISA was performed externally by Guoxin Bio according to the manufacturer’s instructions. Each sample was measured in duplicate. The assay had a sensitivity of 0.094 ng/mL, a detection range of 0.156–10 ng/mL, an intra-assay coefficient of variation (CV) < 8%, and an inter-assay CV <10%. The absorbance was read via a BIO-DL K3 plus microplate reader.

To explore the associations between serum ABCA1 levels and clinical variables, we collected demographic and clinical data from ALS patients. Missing data were addressed via the multiple imputation by chained equations (MICE) method.

(1) Clinical variables with >40% missing data were excluded, retaining 61 clinical features. The missingness proportion for each variable is provided in Table S11 and Figure S7.
(2) Missing values were imputed via MICE with predictive mean matching (method = “pmm”), generating five imputed datasets (m = 5). The imputed datasets are summarized in Table S9.
(3) In each imputed dataset, Spearman correlation was performed between the serum ABCA1 level and each clinical variable. Variables showing *p* < 0.05 in all five imputations were retained. The full summary of correlation coefficients is reported in Table S12, and the post-imputation data distributions are shown in Figure S8.
(4) Four variables were consistently selected: group (ALS vs. control), body mass index (BMI), LDL cholesterol, and weight. The average Spearman’s *r* values across imputations were reported.
(5) A multivariable linear regression model was constructed using serum ABCA1 as the dependent variable and Group, BMI, and LDL as independent predictors. Model performance was summarized by the adjusted R^2^, averaged across the five imputed datasets.
(6) Model assumptions, including linearity, normality of residuals (normal Q‒Q plot), homoscedasticity (scale-location plot), and influence diagnostics (residuals vs. leverage plot), were assessed in the first imputed dataset. Multicollinearity was checked via variance inflation factors.

In addition, functional scores were analyzed to assess clinical relevance. Functional disability was evaluated using the ALS Functional Rating Scale–Revised (ALSFRS-R), and both correlation and group-difference analyses were performed. The complete ALSFRS-R scoring framework is provided in Table S13.

Statistical testing procedures and all regression outputs are comprehensively summarized in Table S10.

#### Spatiotemporal analysis of ABCA1 expression

Publicly available transcriptomic datasets profiling ALS and relevant control samples across peripheral blood, central nervous system regions, and disease stages were obtained from GEO. Dataset accession numbers are listed in the key resources table.

Differential expression was performed using a platform-aware strategy: DESeq2 was applied to count-based RNA-seq datasets, and *limma* was applied to microarray datasets. For DESeq2, analyses were run on raw counts with default size-factor normalization and dispersion estimation; for *limma*, preprocessing followed RMA (background correction, quantile normalization, and log_2_ transformation). Where applicable, *p* values were adjusted for multiple testing as indicated in the figure panels.

Because visualization requires a common scale across datasets, expression values displayed in Figure 8 were taken from VST-normalized data for DESeq2 analyses and from RMA-normalized data for *limma* analyses.

The full set of statistical test results (group contrasts, effect sizes, and *p* values) corresponding to each panel and dataset is compiled in Table S14.

### Quantification and statistical analysis

All analyses were performed in R unless otherwise specified. Statistical tests were chosen according to data distribution and study design, as described in individual method sections. Multiple testing correction was performed using the FDR method when applicable. Figures were generated using the *ggplot2* package. Statistical significance was defined as two-tailed *p* < 0.05 unless otherwise noted. Asterisks in figures indicate the level of statistical significance: ∗*p* < 0.05, ∗∗*p* < 0.01, ∗∗∗*p* < 0.001, and ∗∗∗∗*p* < 0.0001. “ns” denotes not significant.
