# Supplementary material for: ABCA1 acts as a protective modulator in amyotrophic lateral sclerosis
Source: iScience. 2025 Dec 3;29(1):114320. doi: 10.1016/j.isci.2025.114320 (PMC12834111; doi:10.1016/j.isci.2025.114320)
Supplement: Document S1. Figures S1–S8 [file mmc1.pdf]

iScience, Volume *29*

Supplemental information

ABCA1 acts as a protective modulator in amyotrophic lateral sclerosis

Qiang Li, Ge Zhang, Honglin Zheng, Taiqi Zhao, Hang Zhang, Yaochong Zhang, Haiyang Luo, and Yuming Xu

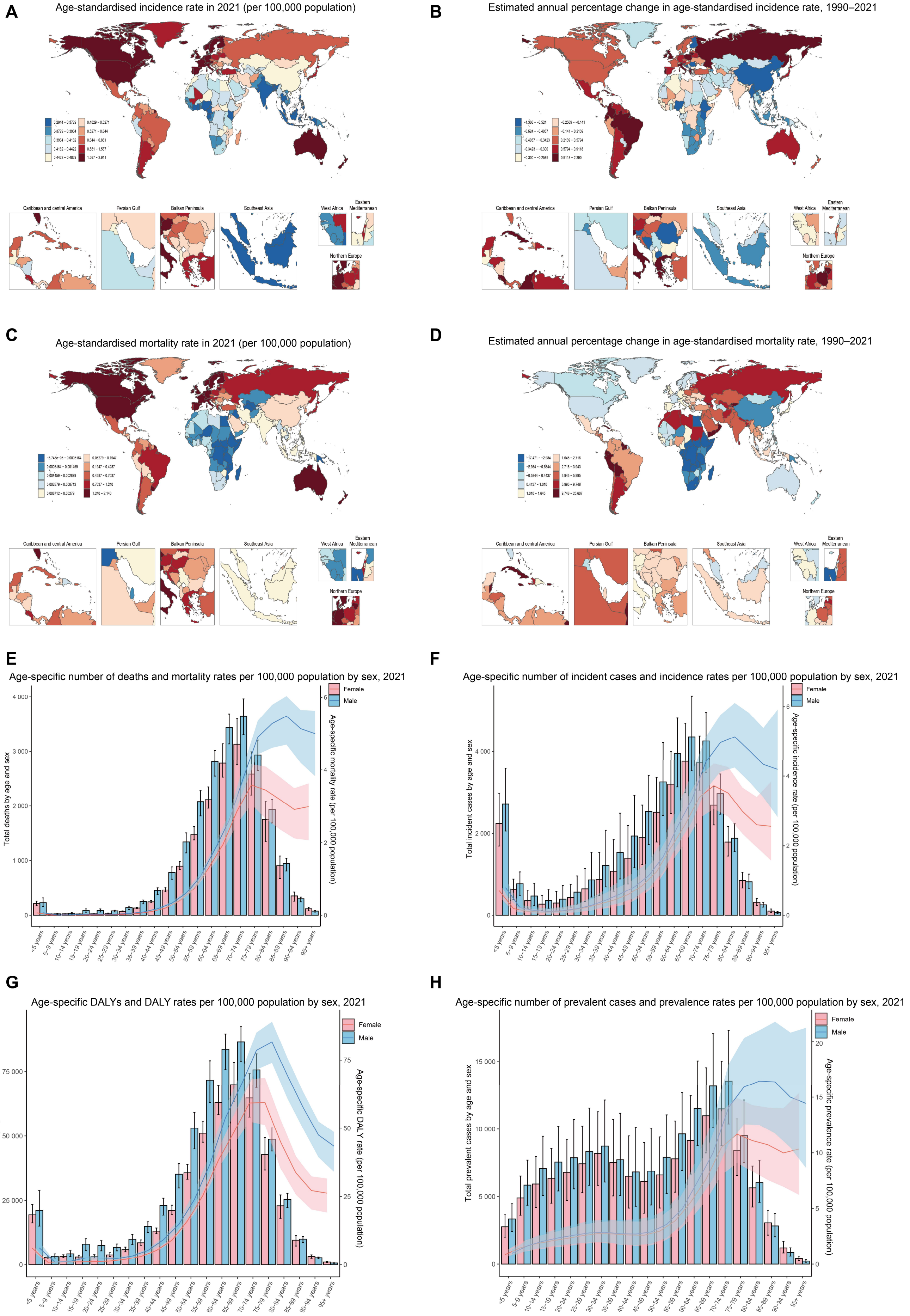
A
Age-standardised incidence rate in 2021 (per 100,000 population)
B
Estimated annual percentage change in age-standardised incidence rate, 1990–2021
C
Age-standardised mortality rate in 2021 (per 100,000 population)
D
Estimated annual percentage change in age-standardised mortality rate, 1990–2021
Caribbean and central America
Persian Gulf
Balkan Peninsula
Southeast Asia
West Africa
Eastern Mediterranean
Northern Europe
E
Age-specific number of deaths and mortality rates per 100,000 population by sex, 2021
Total deaths by age and sex
Age-specific mortality rate (per 100,000 population)
F
Age-specific number of incident cases and incidence rates per 100,000 population by sex, 2021
Total incident cases by age and sex
Age-specific incidence rate (per 100,000 population)
G
Age-specific DALYs and DALY rates per 100,000 population by sex, 2021
Total DALYs by age and sex
Age-specific DALY rate (per 100,000 population)
H
Age-specific number of prevalent cases and prevalence rates per 100,000 population by sex, 2021
Total prevalent cases by age and sex
Age-specific prevalence rate (per 100,000 population)
Female
Male
<5 years
5–9 years
10–14 years
15–19 years
20–24 years
25–29 years
30–34 years
35–39 years
40–44 years
45–49 years
50–54 years
55–59 years
60–64 years
65–69 years
70–74 years
75–79 years
80–84 years
85–89 years
90–94 years
95+ years

Figure S1. Global distribution and age-sex patterns of motor neuron disease (MNDs) burden in 2021.

(A–D) Global maps showing age-standardised rates of MNDs in 2021 and temporal changes since 1990.

(A) Age-standardised incidence rate per 100,000 population in 2021.

(B) Estimated annual percentage change (EAPC) in incidence rate from 1990 to 2021.

(C) Age-standardised mortality rate per 100,000 population in 2021.

(D) EAPC in mortality rate from 1990 to 2021. Sub-regions are shown as insets for selected areas.

(E–H) Age-specific and sex-specific patterns of MNDs burden in 2021.

(E) Number of deaths and mortality rates by age group and sex.

(F) Number of incident cases and incidence rates by age group and sex.

(G) Number of disability-adjusted life years (DALYs) and DALYs rates by age group and sex.

(H) Number of prevalent cases and prevalence rates by age group and sex.

Shaded areas represent 95% uncertainty intervals (UIs) derived from GBD estimation models.

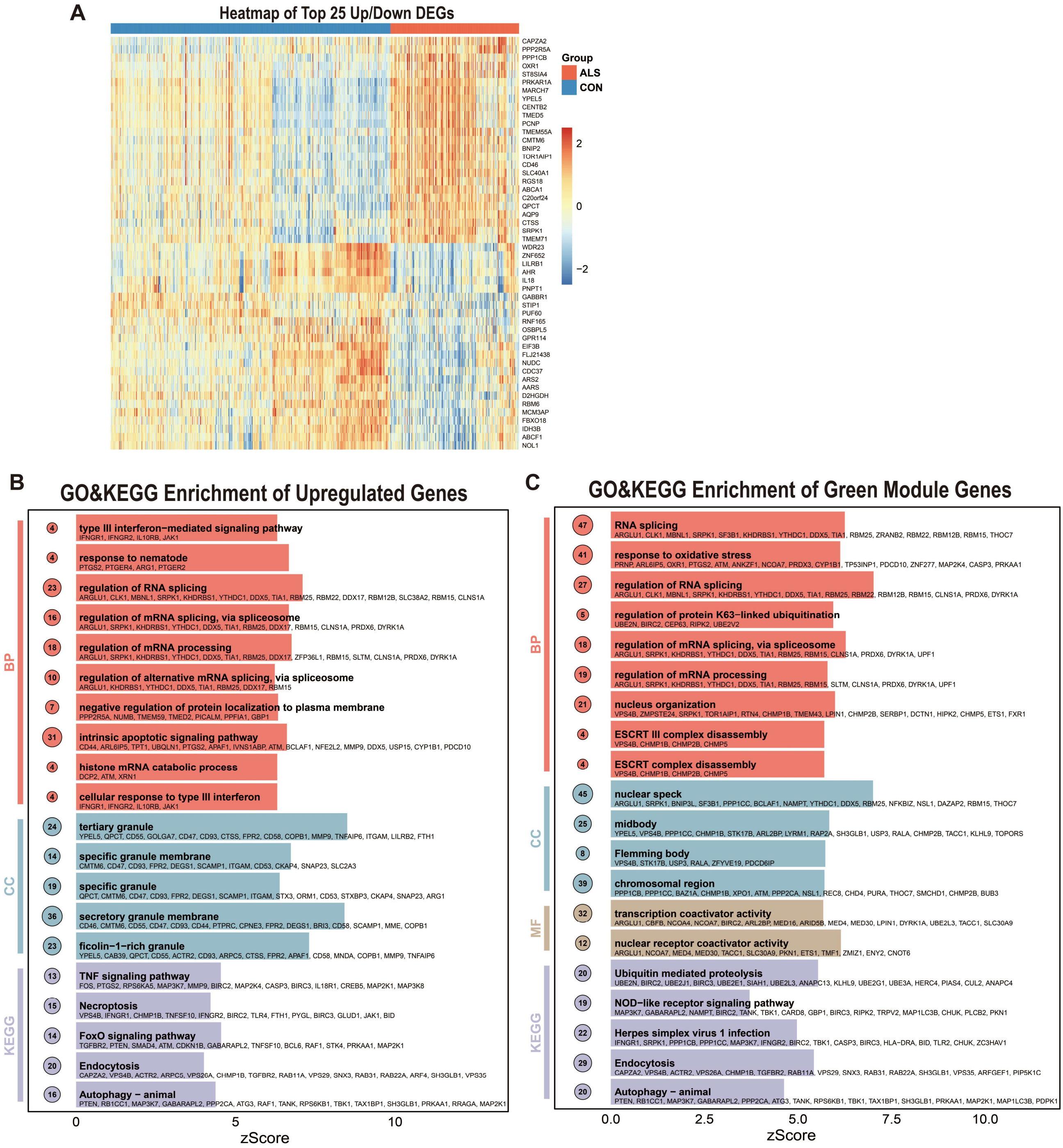

Figure S2. Heatmap and Functional Enrichment of DEGs and ALS-Associated Modules.

(A) Heatmap showing the expression patterns of the top 25 upregulated and top 25 downregulated DEGs across the ALS and control samples, ranked by the FDR.

(B) GO and KEGG pathway enrichment analysis of upregulated genes.

(C) GO and KEGG pathway enrichment analysis of genes from the *green* module.

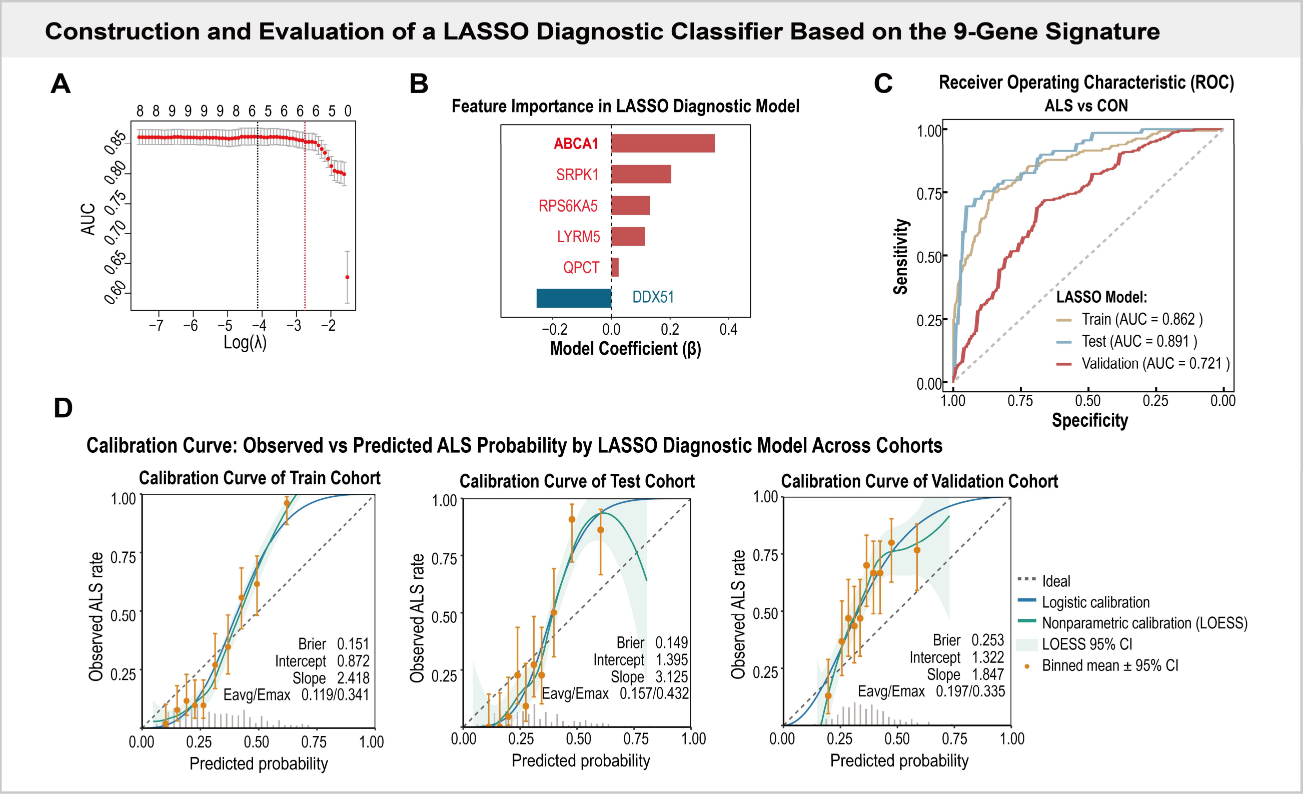

Figure S3. Construction and evaluation of a LASSO diagnostic classifier based on the nine-gene signature.

(A) Cross-validation curve showing the relationship between the area under the receiver operating characteristic curve (AUC) and the regularization parameter (log λ) for the least absolute shrinkage and selection operator (LASSO) model. The dotted line indicates the optimal λ determined by the minimum cross-validated error.

(B) Bar plot of the regression coefficients (β) for the selected genes in the LASSO model. Positive and negative β values represent the direction of association with amyotrophic lateral sclerosis (ALS) diagnosis.

(C) Receiver operating characteristic (ROC) curves of the LASSO classifier for ALS versus controls in the training, test, and validation cohorts.

(D) Calibration curves comparing the predicted and observed ALS probabilities in the three cohorts. Dashed lines represent ideal calibration; solid lines represent logistic and nonparametric (LOESS) calibration. Brier scores, intercepts, and slopes are provided for reference.

Abbreviations: AUC, area under the curve; ROC, receiver operating characteristic; LOESS, locally estimated scatterplot smoothing; ALS, amyotrophic lateral sclerosis; LASSO, least absolute shrinkage and selection operator. Data are presented as mean ± SEM.

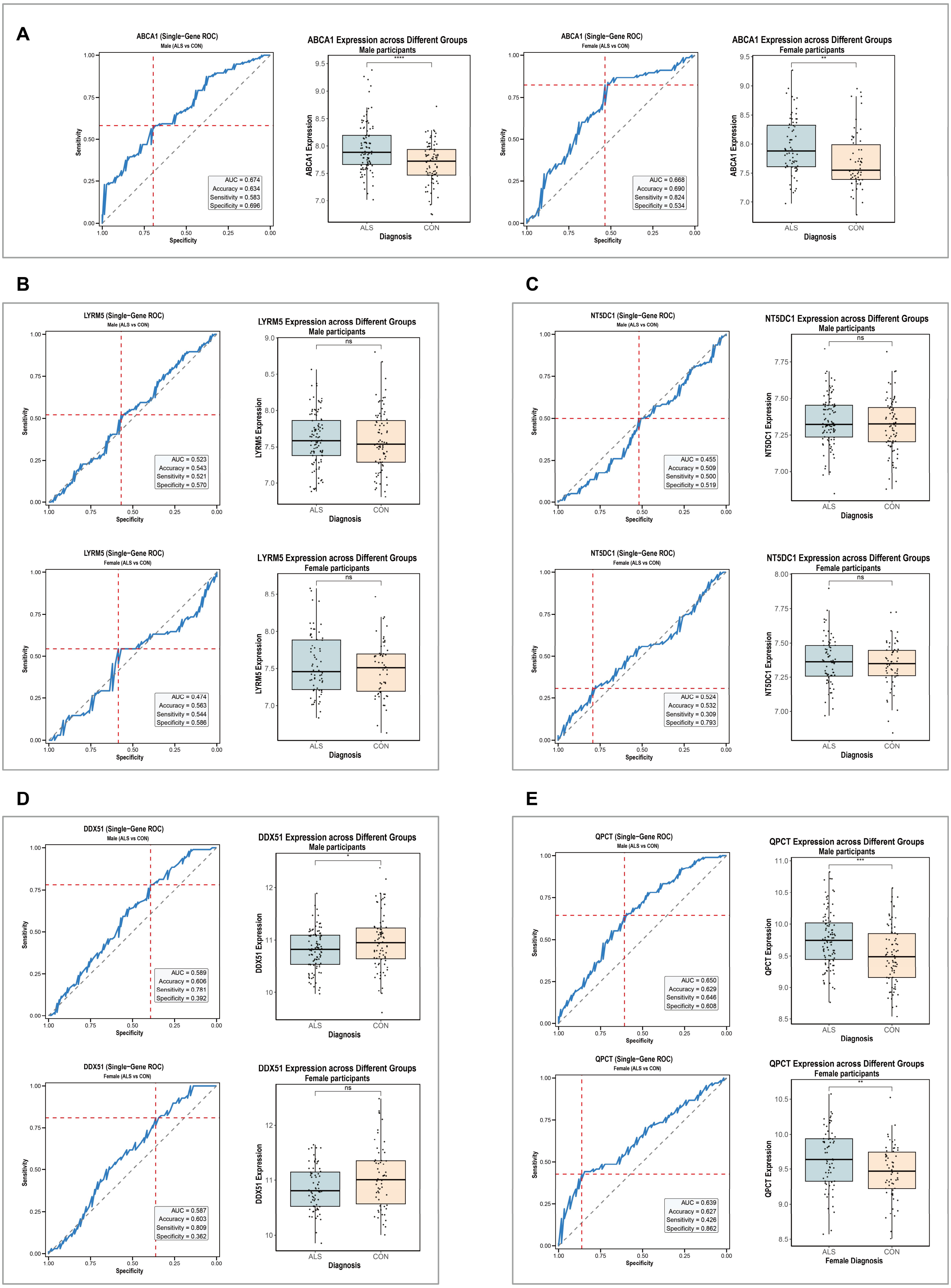

A
ABCA1 (Single−Gene ROC)
Male (ALS vs CON)
AUC = 0.674
Accuracy = 0.634
Sensitivity = 0.583
Specificity = 0.696
ABCA1 Expression across Different Groups
Male participants
****
ABCA1 (Single−Gene ROC)
Female (ALS vs CON)
AUC = 0.668
Accuracy = 0.690
Sensitivity = 0.824
Specificity = 0.534
ABCA1 Expression across Different Groups
Female participants
**
B
LYRM5 (Single−Gene ROC)
Male (ALS vs CON)
AUC = 0.523
Accuracy = 0.543
Sensitivity = 0.521
Specificity = 0.570
LYRM5 Expression across Different Groups
Male participants
ns
LYRM5 (Single−Gene ROC)
Female (ALS vs CON)
AUC = 0.474
Accuracy = 0.563
Sensitivity = 0.544
Specificity = 0.586
LYRM5 Expression across Different Groups
Female participants
ns
C
NT5DC1 (Single−Gene ROC)
Male (ALS vs CON)
AUC = 0.455
Accuracy = 0.509
Sensitivity = 0.500
Specificity = 0.519
NT5DC1 Expression across Different Groups
Male participants
ns
NT5DC1 (Single−Gene ROC)
Female (ALS vs CON)
AUC = 0.524
Accuracy = 0.532
Sensitivity = 0.309
Specificity = 0.793
NT5DC1 Expression across Different Groups
Female participants
ns
D
DDX51 (Single−Gene ROC)
Male (ALS vs CON)
AUC = 0.589
Accuracy = 0.606
Sensitivity = 0.781
Specificity = 0.392
DDX51 Expression across Different Groups
Male participants
*
DDX51 (Single−Gene ROC)
Female (ALS vs CON)
AUC = 0.587
Accuracy = 0.603
Sensitivity = 0.809
Specificity = 0.362
DDX51 Expression across Different Groups
Female participants
ns
E
QPCT (Single−Gene ROC)
Male (ALS vs CON)
AUC = 0.650
Accuracy = 0.629
Sensitivity = 0.646
Specificity = 0.608
QPCT Expression across Different Groups
Male participants
***
QPCT (Single−Gene ROC)
Female (ALS vs CON)
AUC = 0.639
Accuracy = 0.627
Sensitivity = 0.426
Specificity = 0.862
QPCT Expression across Different Groups
Female participants
**
Sensitivity
Specificity
Diagnosis
Female Diagnosis
ALS
CON

F

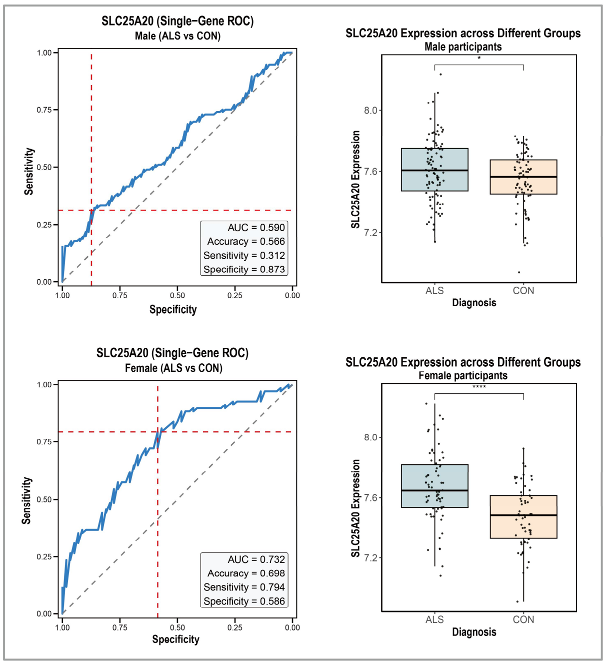
SLC25A20 (Single−Gene ROC)
Male (ALS vs CON)
Sensitivity
Specificity
AUC = 0.590
Accuracy = 0.566
Sensitivity = 0.312
Specificity = 0.873
SLC25A20 Expression across Different Groups
Male participants
SLC25A20 Expression
Diagnosis
ALS
CON
SLC25A20 (Single−Gene ROC)
Female (ALS vs CON)
AUC = 0.732
Accuracy = 0.698
Sensitivity = 0.794
Specificity = 0.586
SLC25A20 Expression across Different Groups
Female participants

G

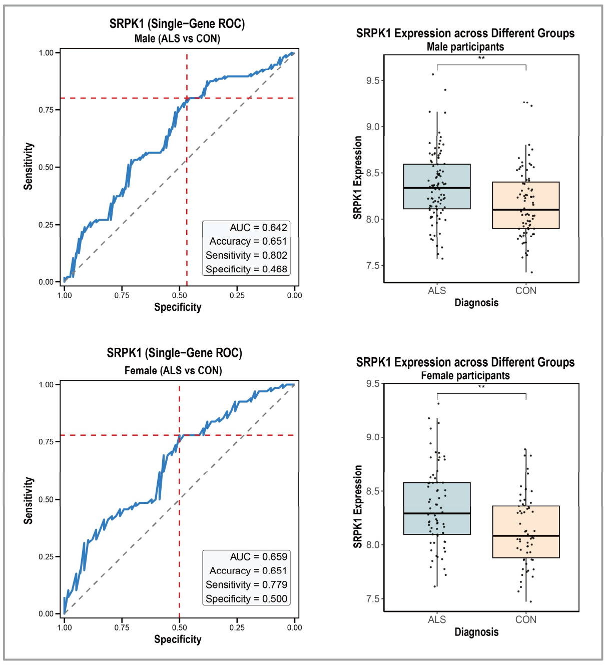
SRPK1 (Single−Gene ROC)
Male (ALS vs CON)
Sensitivity
Specificity
AUC = 0.642
Accuracy = 0.651
Sensitivity = 0.802
Specificity = 0.468
SRPK1 Expression across Different Groups
Male participants
SRPK1 Expression
Diagnosis
ALS
CON
SRPK1 (Single−Gene ROC)
Female (ALS vs CON)
AUC = 0.659
Accuracy = 0.651
Sensitivity = 0.779
Specificity = 0.500
SRPK1 Expression across Different Groups
Female participants

H

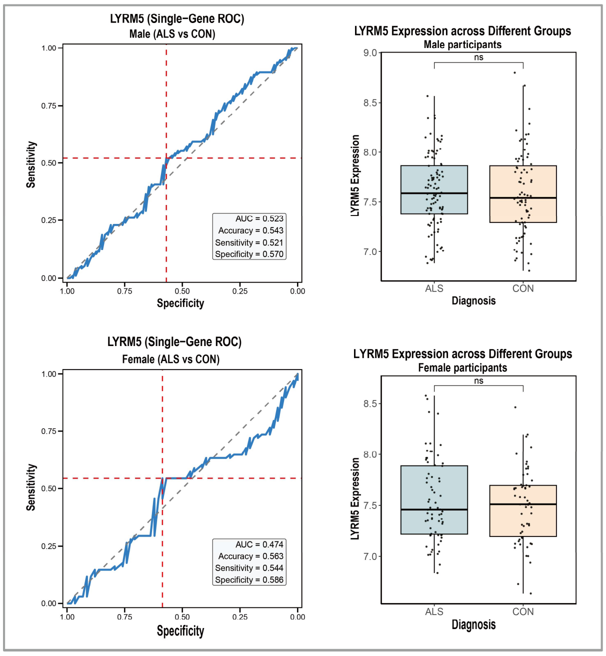
LYRM5 (Single−Gene ROC)
Male (ALS vs CON)
Sensitivity
Specificity
AUC = 0.523
Accuracy = 0.543
Sensitivity = 0.521
Specificity = 0.570
LYRM5 Expression across Different Groups
Male participants
ns
LYRM5 Expression
Diagnosis
ALS
CON
LYRM5 (Single−Gene ROC)
Female (ALS vs CON)
AUC = 0.474
Accuracy = 0.563
Sensitivity = 0.544
Specificity = 0.586
LYRM5 Expression across Different Groups
Female participants

I

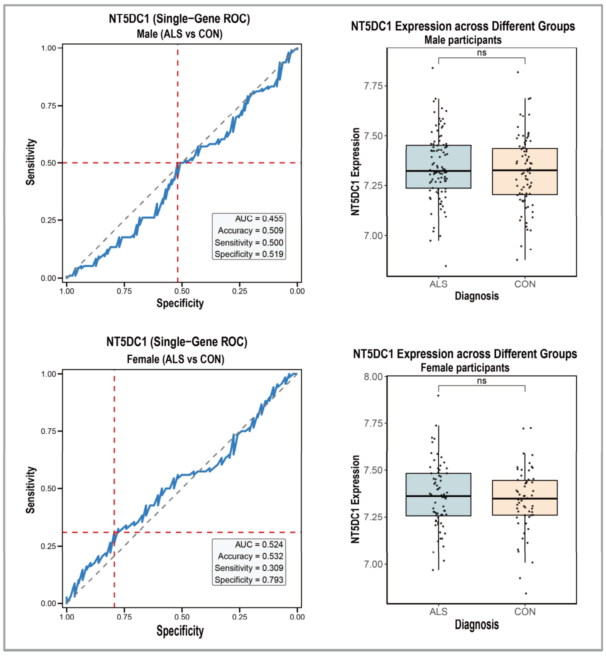
NT5DC1 (Single−Gene ROC)
Male (ALS vs CON)
Sensitivity
Specificity
AUC = 0.455
Accuracy = 0.509
Sensitivity = 0.500
Specificity = 0.519
NT5DC1 Expression across Different Groups
Male participants
ns
NT5DC1 Expression
Diagnosis
ALS
CON
NT5DC1 (Single−Gene ROC)
Female (ALS vs CON)
AUC = 0.524
Accuracy = 0.532
Sensitivity = 0.309
Specificity = 0.793
NT5DC1 Expression across Different Groups
Female participants

Figure S4. Validation of the nine-gene diagnostic signature in the validation cohort with sex-stratified analysis

(A–I) Single-gene receiver operating characteristic (ROC) curves and expression comparisons for each of the nine diagnostic genes in the validation cohort (GSE112680). For each gene, the left panel shows the ROC curve for amyotrophic lateral sclerosis (ALS) versus controls, and the right panel shows box plots of gene expression levels between groups.

ROC curves and box plots are presented separately for male (upper panels) and female (lower panels) participants to evaluate sex-related consistency in diagnostic performance.

Statistical significance of expression differences was assessed using the Wilcoxon rank-sum test.

AUC, accuracy, sensitivity, and specificity values are shown within each ROC panel.

Abbreviations: ALS, amyotrophic lateral sclerosis; ROC, receiver operating characteristic; AUC, area under the curve.

ALS outcome data: Nicolas A (European cohort, ebi-a-GCST005647)

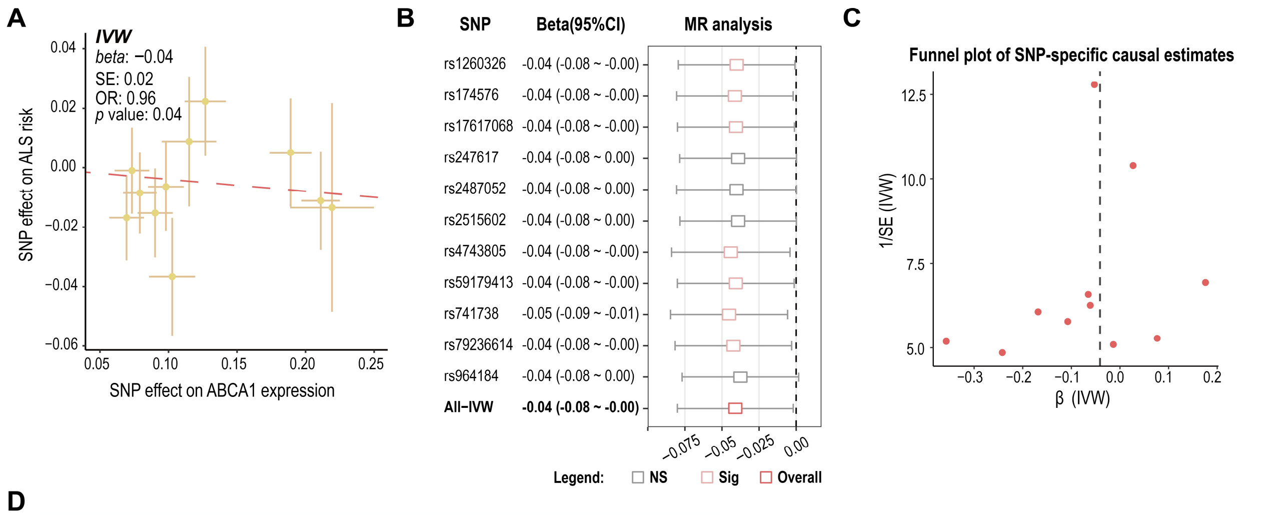

D

MR Method	*β*	OR (95%CI)	*p* value
IVW	-0.04	0.96 (0.92–0.99)	0.040
RAPS	-0.04	0.96 (0.92–1.00)	0.047
IVW-MRE	-0.04	0.96 (0.93–0.99)	0.016
MR Egger	0.05	1.05 (0.95–1.16)	0.344
Weighted median	-0.05	0.95 (0.90–1.00)	0.053
Weighted mode	-0.05	0.96 (0.89–1.03)	0.231

E

Method	*p* value
Directionality – Steiger	$p < 1 \times 10^{-300}$
Pleiotropy - MR Egger (intercept)	0.061
Pleiotropy - MR PRESSO	No outlier
Heterogeneity - MR Egger	0.960
Heterogeneity - IVW	0.913

ALS outcome data: Iacoangeli A (Mixed: European + Chinese, ebi-a-GCST90013429)

F

MR Method	*β*	OR (95%CI)	*p* value
IVW	-0.04	0.96 (0.89–1.04)	0.339
RAPS	-0.04	0.96 (0.89–1.04)	0.315
IVW-MRE	-0.04	0.96 (0.90–1.04)	0.280
MR Egger	0.07	1.07 (0.88–1.29)	0.493
Weighted median	-0.05	0.95 (0.85–1.05)	0.373
Weighted mode	-0.04	0.96 (0.84–1.10)	0.537

G

Method	*p* value
Directionality – Steiger	6.8×10^{-118}
Pleiotropy - MR Egger (intercept)	0.266
Heterogeneity - MR Egger	0.694
Heterogeneity - IVW	0.643

ALS outcome data: Benyamin B (East Asian cohort, ebi-a-GCST004901)

H

MR Method	*β*	OR (95%CI)	*p* value
IVW	-0.01	0.99 (0.97–1.01)	0.333
RAPS	-0.01	0.96 (0.99–1.00)	0.183
IVW-MRE	-0.01	0.99 (0.97–1.01)	0.333
MR Egger	0.01	1.01 (0.96–1.07)	0.685
Weighted median	-0.01	0.99 (0.96–1.01)	0.306
Weighted mode	-0.01	0.99 (0.96–1.02)	0.400

I

Method	*p* value
Directionality – Steiger	1.0×10^{-10}
Pleiotropy - MR Egger (intercept)	0.412
Heterogeneity - MR Egger	0.266
Heterogeneity - IVW	0.281

Figure S5. Mendelian randomization evaluation of ABCA1 expression in relation to amyotrophic lateral sclerosis across three GWAS cohorts

(A–E) European cohort (Nicolas A; ebi-a-GCST005647).

(A) Scatter plot showing single-nucleotide polymorphism (SNP)-specific causal estimates for ABCA1 expression on amyotrophic lateral sclerosis (ALS) risk using the inverse-variance weighted (IVW) method.
(B) Forest plot of leave-one-out analysis, where each point represents the causal estimate obtained after sequentially removing one SNP, with 95% confidence intervals indicating the robustness of the Mendelian randomization (MR) results.
(C) Funnel plot displaying the distribution and symmetry of SNP-specific causal estimates.
(D) Summary table reporting causal estimates obtained from multiple MR methods, including IVW, RAPS, IVW-MRE, MR Egger, weighted median, and weighted mode, with corresponding β, odds ratio (OR), 95% confidence interval (CI), and p values.
(E) Diagnostic statistics for directionality (Steiger test), horizontal pleiotropy (MR Egger intercept, MR-PRESSO), and heterogeneity (MR Egger, IVW).
(F–G) Mixed cohort (European + Chinese; Iacongeli A; ebi-a-GCST90013429).
(F) Summary table of causal estimates derived from the MR methods described above.
(G) Diagnostic statistics for directionality, pleiotropy, and heterogeneity as above.
(H–I) East Asian cohort (Benyamin B; ebi-a-GCST004901).
(H) Summary table of causal estimates derived from multiple MR methods.
(I) Diagnostic statistics for directionality, pleiotropy, and heterogeneity.
Abbreviations: ALS, amyotrophic lateral sclerosis; MR, Mendelian randomization; SNP, single-nucleotide polymorphism; IVW, inverse-variance weighted; OR, odds ratio; CI, confidence interval; RAPS, robust adjusted profile score; IVW-MRE, IVW with multiplicative random-effects; MR-PRESSO, Mendelian randomization pleiotropy residual sum and outlier.
.

Supplementary Results: In-house Validation of Elevated Serum ABCA1 in ALS

A Additional Baseline characteristics of the serum ELISA validation cohort

Characteristic	CON n=15	ALS n=15	p.overall	n
Riluzole Use:			0.100	30
No	15 (100.00%)	11 (73.33%)		
Yes	0 (0.00%)	4 (26.67%)		
LipidDrug Use:			0.100	30
No	11 (73.33%)	15 (100.00%)		
Yes	4 (26.67%)	0 (0.00%)		
Glucose(mmol/L)	5.84 (1.80)	5.57 (1.26)	0.672	26
Creatinine(umol/L)	66.00 [54.50;71.50]	53.00 [45.50;59.00]	0.071	30
Hcy(umol/L)	10.54 [8.41;10.94]	12.34 [10.84;13.30]	0.031	28
Na(mmol/L)	141.00 [139.90;142.00]	140.80 [140.00;142.40]	0.835	30
K(mmol/L)	4.02 (0.48)	3.99 (0.45)	0.858	30
Cl(mmol/L)	104.40 [102.00;105.30]	104.00 [101.15;105.80]	0.648	30
Ca(mmol/L)	2.20 [2.14;2.24]	2.26 [2.23;2.30]	0.055	25
P(mmol/L)	1.04 (0.11)	1.15 (0.16)	0.035	26
AST(U/L)	19.60 (4.93)	28.73 (18.32)	0.081	30
ALP(U/L)	59.86 (24.71)	59.64 (14.46)	0.978	28
DBIL(umol/L)	3.61 [2.99;4.85]	2.90 [2.50;3.30]	0.209	26
IBIL(umol/L)	5.60 [4.00;7.10]	4.90 [3.30;6.80]	0.644	26
ALB(g/L)	41.30 [39.65;43.05]	41.50 [37.93;42.77]	0.597	28
GLB(g/L)	23.00 [21.00;24.60]	24.70 [22.83;27.12]	0.323	28
HB(g/L)	131.00 [126.50;150.00]	133.00 [129.00;144.50]	0.967	30
PLT(e9/L)	187.93 (43.43)	209.47 (68.57)	0.315	30
RBC(e12/L)	4.64 [4.25;4.80]	4.50 [4.26;4.83]	0.901	30
NEUT(e9/L)	3.21 [2.50;4.46]	4.20 [3.20;5.18]	0.254	30
LYM(e9/L)	1.67 [1.39;2.00]	1.78 [1.18;2.02]	0.885	30
MONO(e9/L)	0.37 [0.30;0.46]	0.39 [0.34;0.46]	0.467	30
EOS(e9/L)	0.13 (0.12)	0.16 (0.13)	0.439	30
BASO(e9/L)	0.03 (0.02)	0.03 (0.02)	0.698	30

Values are median [IQR], mean (SD), or n (%). Two-sided overall p values are shown; statistical methods are described in the figure legend.

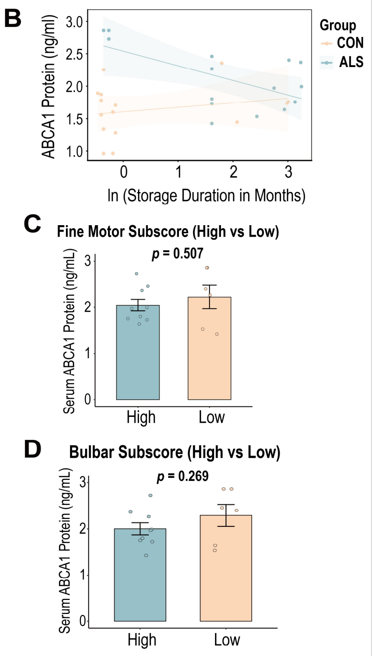

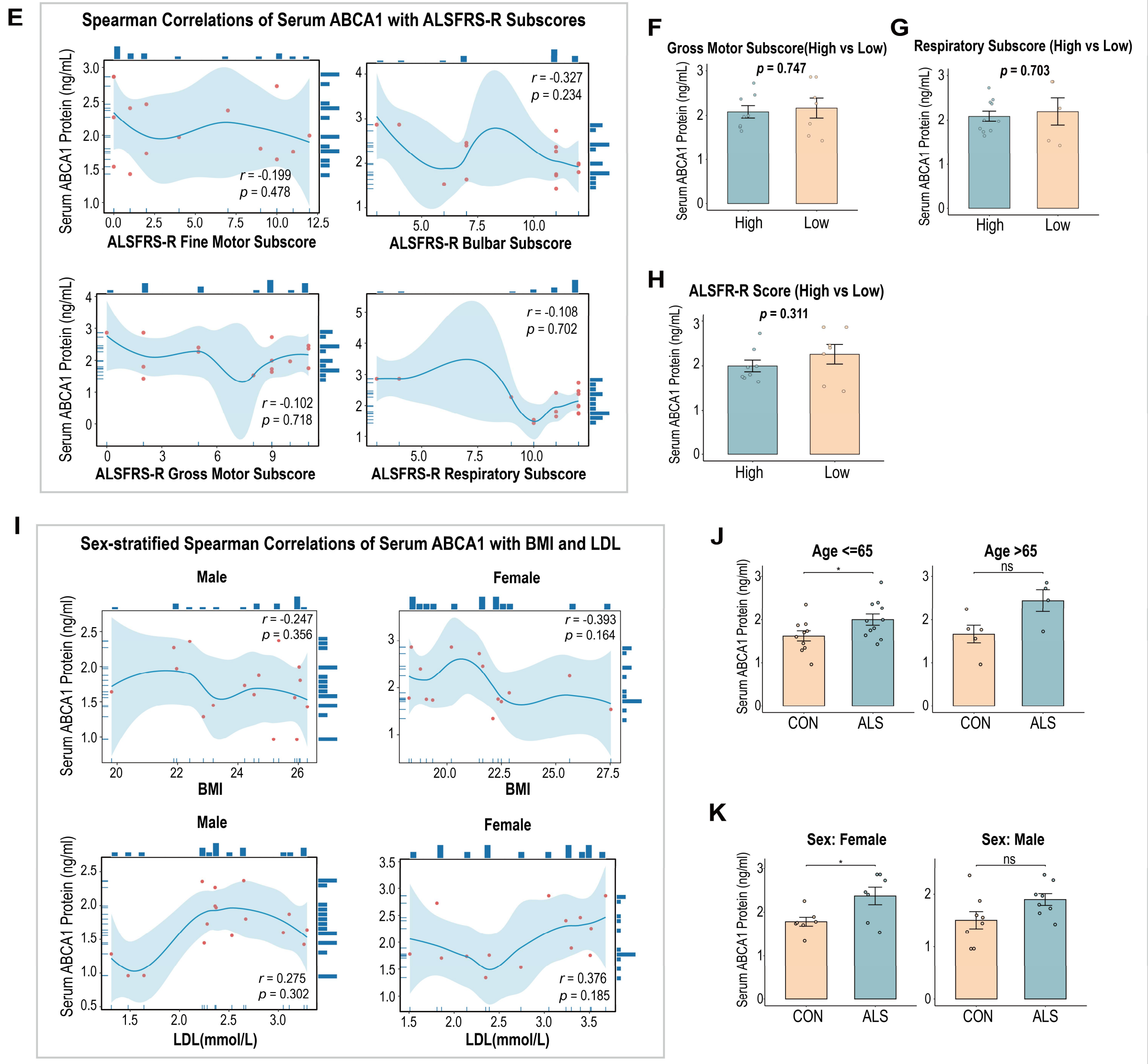

Figure S6. In-house serum validation of ABCA1 expression in amyotrophic lateral sclerosis with extended clinical and stratified analyses

(A) Additional baseline clinical and biochemical characteristics of the serum ELISA validation cohort (ALS, n = 15; Control, n = 15). Data are expressed as median [IQR], mean (SD), or n (%). Two-sided overall p values are shown. Variables include glucose (GLU), creatinine (CREA), homocysteine (Hcy), sodium (Na), potassium (K), chloride (Cl), calcium (Ca), phosphorus (P), alanine aminotransferase (ALT), aspartate aminotransferase (AST), total bilirubin (TBIL), direct bilirubin (DBIL), indirect bilirubin (IBIL), albumin (ALB), globulin (GLB), total protein (TP), platelets (PLT), red blood cells (RBC), neutrophils (NEUT), lymphocytes (LYM), monocytes (MONO), eosinophils (EOS), and basophils (BASO).

(B) Association between serum ABCA1 protein level and sample storage duration.

(C–D) Serum ABCA1 protein levels compared between high and low subgroups of ALSFRS-R fine motor and bulbar function subscores.

(E) Spearman correlations between serum ABCA1 concentration and individual ALSFRS-R subscores (fine motor, bulbar, gross motor, and respiratory).

(F–H) Comparisons of serum ABCA1 protein levels between high and low subgroups of ALSFRS-R gross motor, respiratory, and total scores.

(I) Sex-stratified Spearman correlations of serum ABCA1 with body mass index (BMI) and low-density lipoprotein (LDL) cholesterol levels in male and female participants.

(J–K) Comparisons of serum ABCA1 protein concentrations between ALS and control groups stratified by age (≤65 and >65 years) and by sex (female and male).

Abbreviations: ALS, amyotrophic lateral sclerosis; ELISA, enzyme-linked immunosorbent assay; ALSFRS-R, ALS Functional Rating Scale-Revised; BMI, body mass index; LDL, low-density lipoprotein; GLU, glucose; CREA, creatinine; Hcy, homocysteine; Na, sodium; K, potassium; Cl, chloride; Ca, calcium; P, phosphorus; ALT, alanine aminotransferase; AST, aspartate aminotransferase; TBIL, total bilirubin; DBIL, direct bilirubin; IBIL, indirect bilirubin; ALB, albumin; GLB, globulin; TP, total protein; PLT, platelets; RBC, red blood cells; NEUT, neutrophils; LYM, lymphocytes; MONO, monocytes; EOS, eosinophils; BASO, basophils.

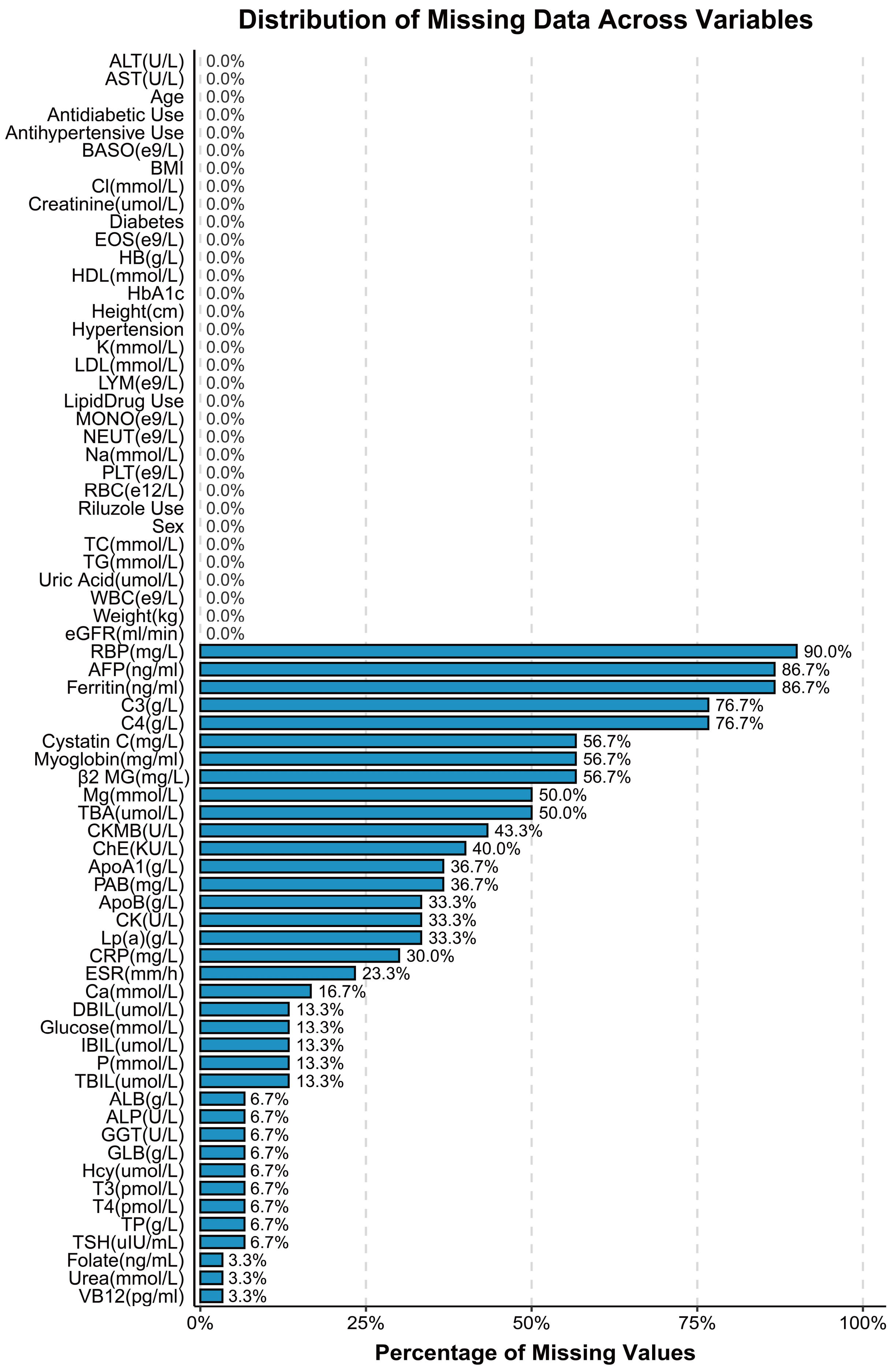

Distribution of Missing Data Across Variables
ALT(U/L) 0.0%
AST(U/L) 0.0%
Age 0.0%
Antidiabetic Use 0.0%
Antihypertensive Use 0.0%
BASO(e9/L) 0.0%
BMI 0.0%
Cl(mmol/L) 0.0%
Creatinine(umol/L) 0.0%
Diabetes 0.0%
EOS(e9/L) 0.0%
HB(g/L) 0.0%
HDL(mmol/L) 0.0%
HbA1c 0.0%
Height(cm) 0.0%
Hypertension 0.0%
K(mmol/L) 0.0%
LDL(mmol/L) 0.0%
LYM(e9/L) 0.0%
LipidDrug Use 0.0%
MONO(e9/L) 0.0%
NEUT(e9/L) 0.0%
Na(mmol/L) 0.0%
PLT(e9/L) 0.0%
RBC(e12/L) 0.0%
Riluzole Use 0.0%
Sex 0.0%
TC(mmol/L) 0.0%
TG(mmol/L) 0.0%
Uric Acid(umol/L) 0.0%
WBC(e9/L) 0.0%
Weight(kg) 0.0%
eGFR(ml/min) 0.0%
RBP(mg/L) 90.0%
AFP(ng/ml) 86.7%
Ferritin(ng/ml) 86.7%
C3(g/L) 76.7%
C4(g/L) 76.7%
Cystatin C(mg/L) 56.7%
Myoglobin(mg/ml) 56.7%
β2 MG(mg/L) 56.7%
Mg(mmol/L) 50.0%
TBA(umol/L) 50.0%
CKMB(U/L) 43.3%
ChE(KU/L) 40.0%
ApoA1(g/L) 36.7%
PAB(mg/L) 36.7%
ApoB(g/L) 33.3%
CK(U/L) 33.3%
Lp(a)(g/L) 33.3%
CRP(mg/L) 30.0%
ESR(mm/h) 23.3%
Ca(mmol/L) 16.7%
DBIL(umol/L) 13.3%
Glucose(mmol/L) 13.3%
IBIL(umol/L) 13.3%
P(mmol/L) 13.3%
TBIL(umol/L) 13.3%
ALB(g/L) 6.7%
ALP(U/L) 6.7%
GGT(U/L) 6.7%
GLB(g/L) 6.7%
Hcy(umol/L) 6.7%
T3(pmol/L) 6.7%
T4(pmol/L) 6.7%
TP(g/L) 6.7%
TSH(uIU/mL) 6.7%
Folate(ng/mL) 3.3%
Urea(mmol/L) 3.3%
VB12(pg/ml) 3.3%
0%
25%
50%
75%
100%
Percentage of Missing Values

Figure S7. Summary of data completeness for all baseline variables.

Horizontal bar chart displaying the percentage of missing values for all analyzed clinical and biochemical variables. Variables are sorted by data completeness, with all variables having 0% missing data grouped at the top. Variables with missing data (>0%) are sorted by descending percentage. The exact percentage of missing values is labeled to the right of each variable.

Abbreviations: ALB, albumin; ALP, alkaline phosphatase; ALT, alanine aminotransferase; ApoA1, apolipoprotein A1; ApoB, apolipoprotein B; AST, aspartate aminotransferase; BASO, basophil count; BMI, body mass index; C3, complement C3; C4, complement C4; Ca, calcium; ChE, cholinesterase; Cl, chloride; CK, creatine kinase; CKMB, creatine kinase-MB; CRP, C-reactive protein; DBIL, direct bilirubin; eGFR, estimated glomerular filtration rate; EOS, eosinophil count; ESR, erythrocyte sedimentation rate; GGT, gamma-glutamyl transferase; GLB, globulin; Hb, hemoglobin; HbA1c, hemoglobin A1c; Hcy, homocysteine; HDL, high-density lipoprotein cholesterol; IBIL, indirect bilirubin; K, potassium; LDL, low-density lipoprotein cholesterol; Lp(a), lipoprotein(a); LYM, lymphocyte count; Mg, magnesium; MONO, monocyte count; Na, sodium; NEUT, neutrophil count; P, phosphorus; PAB, prealbumin; PLT, platelet count; RBC, red blood cell count; RBP, retinol-binding protein; T3, triiodothyronine; T4, thyroxine; TBA, total bile acid; TBIL, total bilirubin; TC, total cholesterol; TG, triglycerides; TP, total protein; TSH, thyroid-stimulating hormone; VB12, vitamin B12; WBC, white blood cell count.

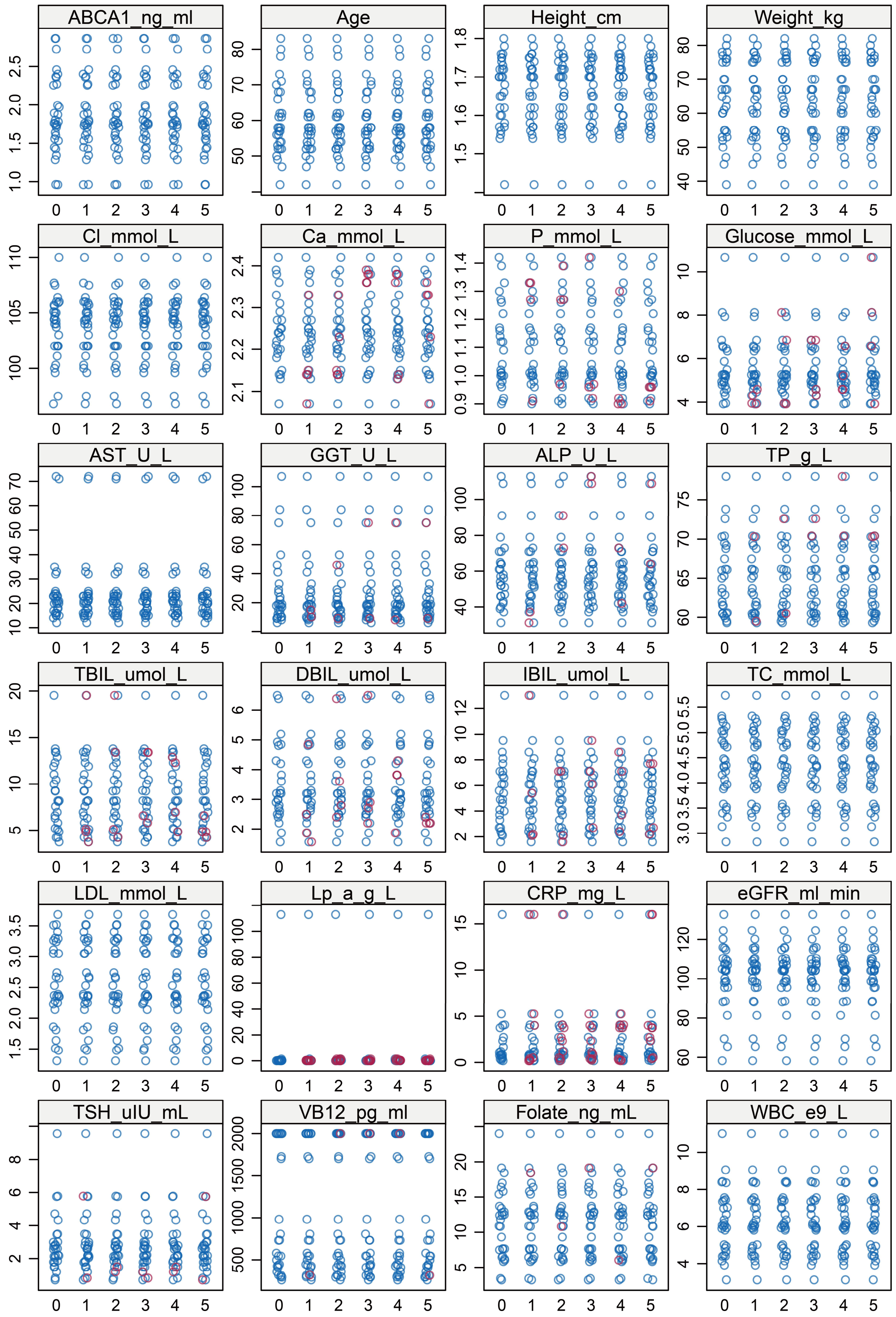
ABCA1_ng_ml
Age
Height_cm
Weight_kg
Cl_mmol_L
Ca_mmol_L
P_mmol_L
Glucose_mmol_L
AST_U_L
GGT_U_L
ALP_U_L
TP_g_L
TBIL_umol_L
DBIL_umol_L
IBIL_umol_L
TC_mmol_L
LDL_mmol_L
Lp_a_g_L
CRP_mg_L
eGFR_ml_min
TSH_uIU_mL
VB12_pg_ml
Folate_ng_mL
WBC_e9_L

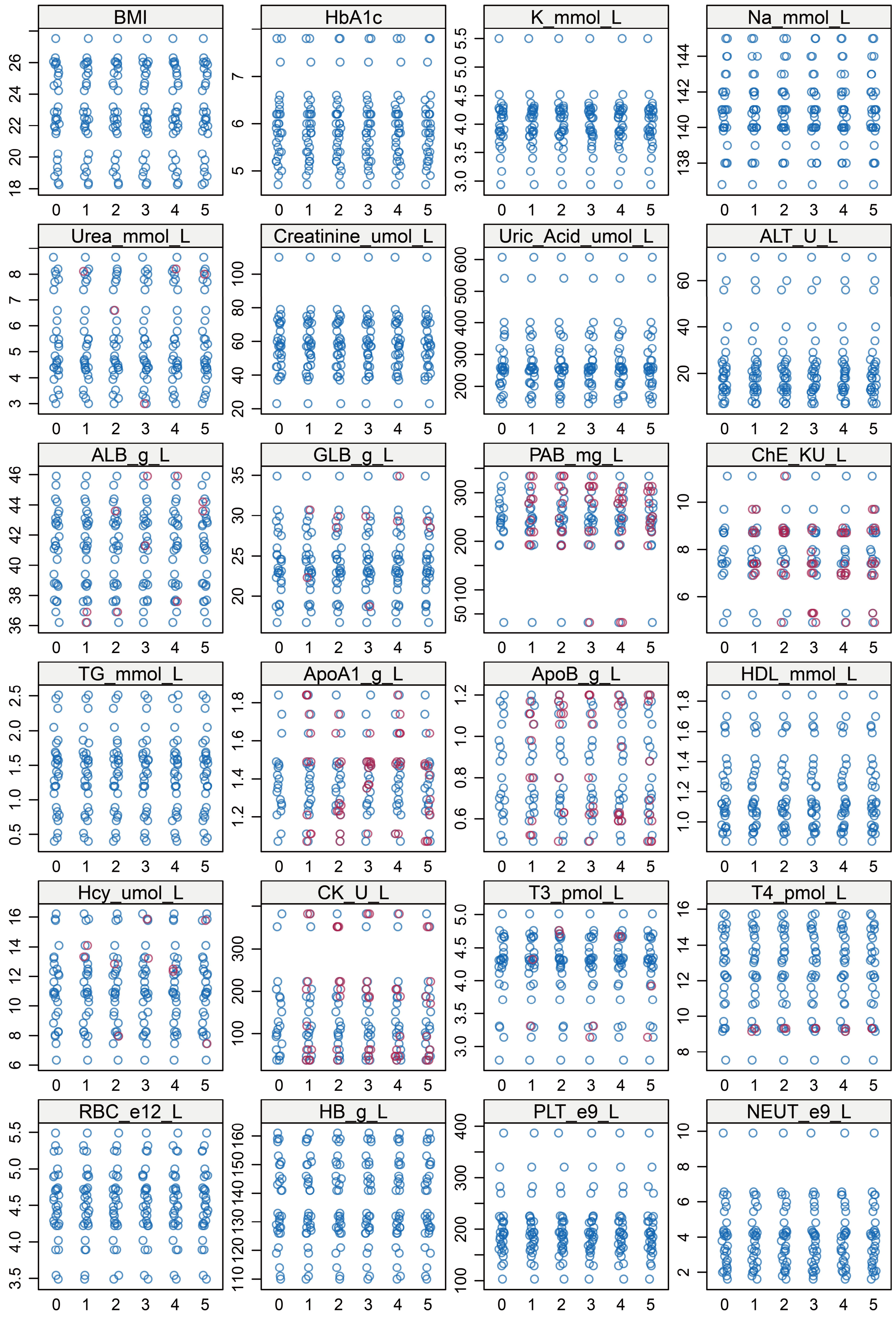
BMI
HbA1c
K_mmol_L
Na_mmol_L
Urea_mmol_L
Creatinine_umol_L
Uric_Acid_umol_L
ALT_U_L
ALB_g_L
GLB_g_L
PAB_mg_L
ChE_KU_L
TG_mmol_L
ApoA1_g_L
ApoB_g_L
HDL_mmol_L
Hcy_umol_L
CK_U_L
T3_pmol_L
T4_pmol_L
RBC_e12_L
HB_g_L
PLT_e9_L
NEUT_e9_L
0 1 2 3 4 5

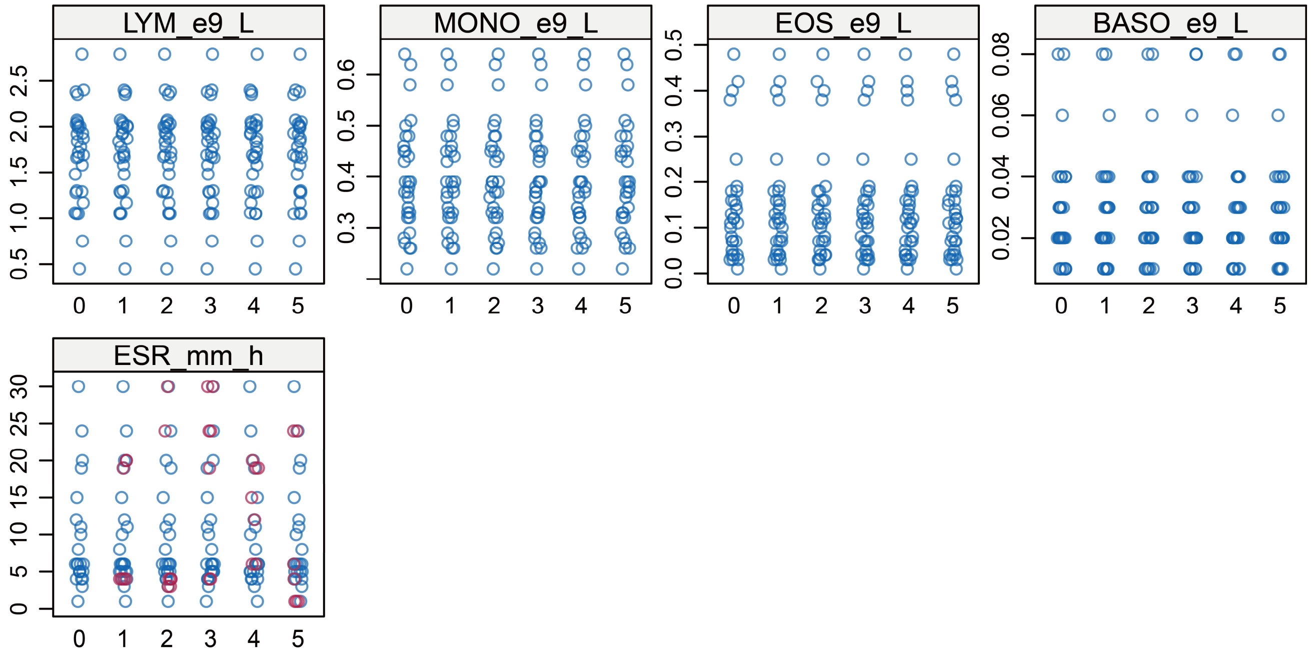

Figure S8. Distribution of clinical and biochemical variables across multiple imputed datasets after MICE imputation

Strip plots showing the distribution of demographic, clinical, and biochemical variables across five imputed datasets generated by multiple imputation using chained equations (MICE).

Each point represents an individual observation from one of the five imputed datasets (m = 1–5).

Blue circles indicate original observed values, and red circles indicate imputed values generated during the MICE procedure.

This visualization allows assessment of distributional consistency and plausibility of imputed data across all variables.

Variables include:

Demographic and anthropometric indicators: age, height, weight, body mass index (BMI), and hemoglobin A1c (HbA1c).

Electrolytes and renal function: sodium (Na), potassium (K), chloride (Cl), calcium (Ca), phosphate (P), urea, creatinine (CREA), uric acid (UA), and estimated glomerular filtration rate (eGFR).

Liver function: aspartate aminotransferase (AST), alanine aminotransferase (ALT), gamma-glutamyl transferase (GGT), alkaline phosphatase (ALP), total protein (TP), albumin (ALB), globulin (GLB), prealbumin (PAB), total bilirubin (TBIL), direct bilirubin (DBIL), and indirect bilirubin (IBIL).

Lipid metabolism: total cholesterol (TC), triglycerides (TG), high-density lipoprotein cholesterol (HDL), low-density lipoprotein cholesterol (LDL), lipoprotein(a) [Lp(a)], apolipoprotein A1 (ApoA1), and apolipoprotein B (ApoB).

Endocrine and nutritional markers: thyroid-stimulating hormone (TSH), triiodothyronine (T3), thyroxine (T4), vitamin B12 (VB12), and folate.

Inflammatory and metabolic indicators: C-reactive protein (CRP), homocysteine (Hcy), creatine kinase (CK), and erythrocyte sedimentation rate (ESR).

Hematological indices: hemoglobin (HB), red blood cell count (RBC), platelet count (PLT), white blood cell count (WBC), neutrophil count (NEUT), lymphocyte count (LYM), monocyte count (MONO), eosinophil count (EOS), and basophil count (BASO).

Other biochemical indicators: cholinesterase (ChE).

The comparable value ranges between red (imputed) and blue (observed) points across datasets confirm the

reliability and distributional stability of the MICE imputation.

Abbreviations: MICE, multivariate imputation by chained equations; BMI, body mass index; HbA1c, hemoglobin A1c; AST, aspartate aminotransferase; ALT, alanine aminotransferase; GGT, gamma-glutamyl transferase; ALP, alkaline phosphatase; TP, total protein; ALB, albumin; GLB, globulin; PAB, prealbumin; TBIL, total bilirubin; DBIL, direct bilirubin; IBIL, indirect bilirubin; TC, total cholesterol; TG, triglycerides; HDL, high-density lipoprotein cholesterol; LDL, low-density lipoprotein cholesterol; Lp(a), lipoprotein(a); ApoA1, apolipoprotein A1; ApoB, apolipoprotein B; TSH, thyroid-stimulating hormone; T3, triiodothyronine; T4, thyroxine; VB12, vitamin B12; CRP, C-reactive protein; Hcy, homocysteine; CK, creatine kinase; ESR, erythrocyte sedimentation rate; HB, hemoglobin; RBC, red blood cell count; PLT, platelet count; WBC, white blood cell count; NEUT, neutrophil count; LYM, lymphocyte count; MONO, monocyte count; EOS, eosinophil count; BASO, basophil count; ChE, cholinesterase; eGFR, estimated glomerular filtration rate.
